# Health care use as an aspect of immigrant integration? An analysis of health care cost convergence among new immigrants and natives in Finland

**DOI:** 10.1016/j.jmh.2025.100386

**Published:** 2025-12-11

**Authors:** Maria Vaalavuo, Tuukka Holster, Natalia Skogberg, Heidi Kuusinen

**Affiliations:** Finnish Institute for Health and Welfare, PL 30, 00271 Helsinki, Finland

**Keywords:** Immigration, Integration, Health care costs, Health care use, Healthy immigrant effect, Register data

## Abstract

The aim of this study is to analyse 1) how average health care costs differ between newly arrived immigrants and natives in Finland, 2) whether the costs of immigrants converge to the level of natives over time, and 3) how other factors of integration are associated with convergence in health care use. We use individual-level register data on total working-age (18–64) population living in Finland between 2008 and 2017 combined with their health care use in public specialized health care. We focus on immigrants who arrived in Finland between 2008–2010. To illustrate trajectories in health care costs, we employ growth curve models. Our results show that immigrants have lower health care costs compared to natives: on average native costs are 1.6 times higher than those of immigrants. Moreover, they do not converge to the native level over a 7-years observation period. This finding of little convergence over time holds also among immigrants who have more local social, cultural, and economic capital, although the native-immigrant gap is narrower among more integrated immigrants. Notably, the costs differ remarkably between different immigrant groups and by other factors. Information on these differences is crucial for assessing equity in the distribution of health care. In addition to better health among immigrants, lower health care use among immigrants may indicate, for example, different approaches to health care use or unmet needs due to barriers to accessing health care services. To make informed policy decisions, future research is needed to uncover the factors behind the lower health care use among immigrants and whether this affects health outcomes and health inequality.

## Highlights

• Health care use and costs of recent immigrants remain lower compared to native Finnish population over time.
• Accounting for other measures of integration narrows the gap between immigrants and natives, but convergence over time remains modest among more integrated immigrants too.
• Significant differences in health care costs exist between immigrant groups.
• In addition to better health, the results can indicate unmet health care needs among immigrants.
• In the context of increasing immigration, improving access to health care among immigrants is important for ensuring health equity.

## 1 Introduction

Alongside an aging population, one of the most prominent factors affecting the population structure in European welfare states is immigration. Although net migration into Finland, a Nordic welfare state, is a relatively new phenomenon, the number of immigrants has steadily grown since the 1990′s. Today 9.6 per cent of the Finnish population has been born abroad (Statistics Finland, 2023). In the context of increasing diversity, understanding differences in the determinants of health care use and improving access to health care among immigrants is important for ensuring health equity. In Finland, the Health Care Act (1326/2010) aims to reduce health inequalities between population groups and to ensure equal access, quality, and patient safety. Still, significant disparities have been observed in both health outcomes and health care use based on socioeconomic status and other factors (e.g., van Doorslaer et al., 1997; Huijts et al., 2010; Vaalavuo, 2020).

Immigrants in Finland are a heterogenous group, showing both health advantages and disadvantages in health compared with the general Finnish population. On average, immigrants in Finland have a lower prevalence of self-reported long-term illnesses (Laatikainen et al., 2020). Other studies have found that the prevalence of psychological distress and diabetes is generally higher among immigrants compared to the general population (Skogberg et al., 2018; Robertsson et al., 2023). It is also important to note that pronounced differences in health tend to emerge when data is disaggregated by region of origin, with particularly immigrants of Middle Eastern and African origin overrepresented among those with greater health problems (Rask et al., 2016; Skogberg et al., 2016, 2018; Robertsson et al., 2023).

Existing studies have found that immigrants generally have lower health care use and costs compared to the majority population (e.g., Wilson et al., 2020; Çilenti et al., 2021; Kieseppä et al., 2020; Xu et al., 2021; Barlow et al., 2022). While some studies propose this may be due to inequality in access to health care (supply-related factors), other studies suggest it is due to lower overall need for these services (demand-related factors). The latter explanation draws from the literature on the “healthy immigrant effect” (HIE) that posits that immigrants tend to be healthier than individuals from both the receiving country and the country of origin as selection occurs, with those who are healthier being more likely to migrate and to stay in the destination country (e.g., McDonald and Kennedy, 2004; Kennedy et al., 2015; Holz, 2022; Hagos and Hamilton, 2024). The health effect is especially observed in the immediate years after migration, while “acculturation” is argued to result in smaller native-immigrant health differences in the longer term (Hagos and Hamilton, 2024). In fact, the “weathering” hypothesis argues that immigrants experience a more rapid health decline with age (Loi et al., 2025). However, a recent study from Finland argues that the HIE continues until old age with evidence on migrant mortality advantage across various causes of death (Kemppainen et al., 2024).

The HIE theory has also been contested as it oversimplifies an intricate, complex, and multidimensional phenomenon (e.g., Vang et al., 2016; Helgesson et al., 2019; Elshahat et al., 2022). It seems unlikely that health is the only factor causing differences in health care use. The country of origin, the conditions in the country of origin as well as in the country of destination, the reason for migration, and the process of immigration and integration all contribute to immigrants’ health and health care use in addition to the typical factors affecting health care use and social determinants of health. Previous research has also highlighted multiple barriers in immigrants’ access to health services, such as language, unsatisfactory experiences, inadequate knowledge about available services and perceptions about where and when to seek care (Akhtar et al., 2022; Pandey et al., 2022; Arsenijevic and Seibel, 2024). Consequently, achieving equal access and use of services as well as equal outcomes of health care use may require a high level of sociocultural integration from immigrants, as navigating the health care system can prove to be difficult even among the natives. Various forms of social, cultural, and economic capital are necessary to understand how the health care system works, and what services are available (Willis et al., 2016; Straiton and Myhre, 2017).

Our study combines research interests and wider societal concerns about health care costs, immigration integration, and health inequality. The aim is to study how health care use and costs differ among immigrants and native population in Finland. Using rich register data and following health care costs in public specialized health care from 2011 to 2017, we ask:

1) How do average health care costs per capita in public specialized health care sector differ between newly arrived immigrant groups and natives in Finland?
2) Do costs among the immigrants converge to the level of natives over the 7-year observation period?
3) How do the individual health care cost trajectories vary between different immigrant groups and over time?
4) Does integration in other areas foster convergence of health care costs between immigrants and natives?

It is important to look at the health care use and costs among immigrants. In this study, we approach the convergence of health care costs between immigrants and natives as a potential indicator of integration within the health care system. First, it may reflect integration in the service system of the country of migration, which we regard to be an important aspect of integration. However, we acknowledge that convergence in costs is a complex indicator, which does not necessarily indicate successful integration. It may reflect changes in health status (e.g., health deterioration or “weathering”), growing familiarity with the health care system, or improved access to services. Therefore, we interpret cost convergence cautiously and discuss it alongside alternative explanations such as acculturation processes, differences in unmet health care needs, and varying health trajectories after migration. We also study cost convergence in relation to other dimensions of integration. Second, it enhances our understanding of how equitable the health care system is. While accounting for care needs is beyond the scope of this study and not possible with the data used here, we acknowledge these as alternative interpretations. Third, better understanding of health care use and its trajectory among the immigrant population may serve as an important tool in developing further the models used to predict the future health expenditures at the population level. Fourth, if an increasing proportion of the population will consist of immigrants, understanding their service use is necessary for developing a culturally competent and accessible system.

We compare use of health care of immigrants to natives in Finland using data for the years 2008–2017 and a rich set of Finnish register data. Our data includes the full population of Finland and for almost every individual, information on the country of origin and the date when the person moved to Finland (or more precisely acquired a home municipality in Finland). The data also includes comprehensive individual-level information on health care utilization, demographics, and socioeconomic background. Information on socioeconomic background includes income, labour market status, marital status, and family members. We are then able to build on the previous literature by having much more detailed information on health care use over time and rich set of background variables, for the entire immigrant cohort that arrived in Finland between 2008–2010.

Our most important contribution to the literature is that we follow health care use of immigrants for several years after moving to Finland to see if it converges to that of natives. Comparing mean health care use of immigrants to the native-born answers if, on average, immigrants use services as much as the native population. While analysing average differences is important, for many policy questions it is more relevant to know whether differences between immigrants and natives tend to persist: it could be that differences in mean health care use are driven by lower use one or two years after the move, after which immigrants’ cost levels converge to those of the natives. Such short-term differences would seem less likely to warrant further research or to require interventions to increase access to health care. We use growth curve models to analyse changes in health care use over time. Growth curves allow us to flexibly characterize the development of health care use after migration to Finland and to compare the curves between immigrants and native-born Finns.

We also analyse how the trajectory of health care use depends on integration in other areas. “Integration in other areas” refers to proxies of local social capital, namely having a Finnish spouse, a workplace with dominantly native-born co-workers and neighbourhood’s ethnic composition, and socioeconomic status (employment and income status). These can be expected to provide the forms of social, cultural, and economic capital that are needed to navigate the health care system. Finally, we extend the analyses to immigrant cohorts that have arrived in Finland earlier to see whether there is a pattern of convergence when immigrants have lived in Finland for much longer.

## 2 Literature and theoretical background

### 2.1 Health care use by immigrants and demand and supply of health care

Health and health care use are affected by various sociocultural, socioeconomic, and demographic factors both in the immigrant and native populations. Andersen’s conceptual framework conceives the use of health care as a function of predisposing, enabling, and need factors (Andersen and Newman, 1973). The predisposing factors include sociodemographic variables, such as age and gender, educational level, and income, that are associated with health care needs and morbidity. Need factors include health status and how it is perceived by the individual and medical professionals. Enabling factors include not only economic resources that allow access to services but also information on the available services and other factors associated with accessibility and availability of services. Enabling factors then include factors that in the economics literature would be called supply-factors, such as the number of local service providers and the distance to them. Enabling factors also include some demand-factors, such as income, but most demand-factors would be predisposing and need variables. Immigrants may use less health care for reasons related to both supply and demand of services.

The healthy immigrant effect (HIE) is a widely studied phenomenon in the literature on immigrant health. According to the healthy immigrant effect, lower health care use by immigrants is driven by selection: those in better health tend to have a higher propensity to migrate and stay in the destination country. If this is the case, some, or all of the observed difference in average health care use is the result of differences in health. However, the evidence is mixed. The theory suggests that immigrants tend to have better health outcomes compared to the native population, particularly in the early period after their arrival in a new country. This effect has been supported by evidence from various countries (e.g., Kennedy et al., 2015; Helgesson et al., 2019; Holz, 2022; Ichou and Wallace, 2019). A recent study by Kemppainen et al. (2024) argued that immigrants in Finland have a mortality advantage also among those aged 70 years and older, but the study did not follow individuals over time to see whether there is any convergence or what is often called “acculturation” in terms of health (Hagos and Hamilton, 2024).

While evidence in support of the theory is abundant, some studies have found contrasting results, indicating that immigrants’ health may be poorer than that of the native population, particularly in European contexts (see e.g., Iglesias et al., 2003; Solé-Auró and Crimmins, 2008). Additionally, the validity of the HIE may vary depending on the origin of the immigrants. For instance, Helgesson et al. (2019) observed evidence of the HIE among Western immigrants but not among non-Western immigrants. Conversely, Kennedy et al. (2015) found that the HIE is stronger for immigrants from developing countries than for those from developed countries. It is questionable to what extent HIE can explain health care use for the heterogeneous immigrant group as a whole; therefore, a more intricate analysis is necessary both for testing the hypothesis as well as for any solid policy implications (see also Domínguez-Rodríguez and González-Rábago, 2024).

Other potential demand-side factors affecting health care use include economic resources that vary greatly between immigrants and natives also long after arrival to the host country (see Vaalavuo and Rask, 2024, on earnings trajectories of immigrants in Finland). Income may affect service use especially if there are high monetary costs for accessing care; this is not really the case in Finland where user fees in public health care were moderate in the study period, while unmet needs due to economic burden have also been reported (e.g., Aaltonen and Vaalavuo, 2024). Preferences of care intensity refer to the attitudes and information concerning the benefits and inconveniences of health care interventions. Information may also include the simple knowledge of what kind of services are available and what individuals are entitled to receive. Knowledge on the service system, health literacy, experiences of racism and discrimination, and trust in the health care system and professionals could be especially important demand-side factors for individuals with a foreign background, while also stigma related to service use could also explain differences in health care use among some immigrant groups and natives. While a number of studies report an association between experiences or anticipation of discrimination with seeking or foregoing health services (Rivenbark and Ichou, 2020), some studies have, in contrast, found an increase in health service use or no associations (Gazard et al., 2018; Dehkordy et al., 2016).

Data on preferences, health literacy, discrimination, or knowledge is not available in registers, but variables like having a Finnish spouse or living in a neighbourhood / working in a workplace with a majority of natives can be used as proxies for the information set of the individual. In general, we expect that integration in other aspects is related to health care use of immigrants being more similar to the native levels – a hypothesis that we test in this article.

**Fig. 1:**
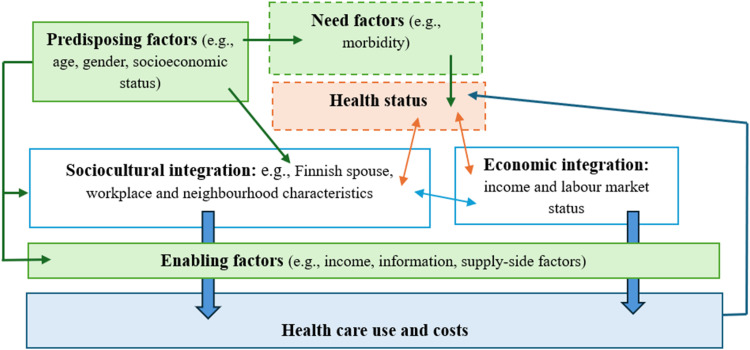
Conceptual framework of the study. Note: The dashed lines in the boxes on need factors and health status indicate that we do not have information on these in our data.

Fig. 1 illustrates the conceptual framework of our study, focusing on the potential factors affecting health care use and costs among immigrants and linking the above-mentioned factors to the integration process. We will not empirically test the different causal pathways outlined in this figure, but the figure aims to capture the relations between various factors and integration as well as potential health selection in other integration outcomes.

### 2.2 The Finnish context

In Finland, health care is largely publicly financed, organized, and produced. Primary care and specialized medicine are mostly organized and produced by local authorities, but public health care is complemented with an occupational health care system and private care. Occupational health care and private care consist mostly of ambulatory primary care but may give referrals to public specialized medicine. All residents are entitled to use public care, but access to occupational health care depends on being employed and services covered differ between employers. Occupational health care is mainly produced by private firms and is paid for by payroll deductions from employers and employees. There is a National Health Insurance system that partly reimburses the use of private care, but the subsidies are small, and access is therefore largely determined by the ability to pay. Outpatient drugs are not provided by local authorities and are instead reimbursed by the National Health Insurance. Unfortunately, for our study period there is no comprehensive register data available for use of public primary care, occupational health care and private health care. Because of this, we focus on specialized care which is almost completely produced by the public sector. In Finland, the specialized care amount to around two-thirds of all public health care costs. During the study period, the local authorities financed health care by a combination of local taxation, funding from the state and relatively low user fees with an annual ceiling. Because of autonomy and the differences in ability to raise taxes, there are geographical differences in supply. These are partly taken into account by including information on the region of residence in the models.

## 3 Data and methods

### 3.1 Data and analysis sample

We utilize various linked Finnish register data sources. The data combine rich background information on the total population of Finland from Statistics Finland and information on health care use and costs from the Finnish Institute for Health and Welfare (THL). The data are put together by the INVEST Flagship Research Centre.^1^ The data include the total population residing permanently in Finland. A 10 per cent random sample of Finns was drawn for the analyses for analytical feasibility, while the immigrant population includes all immigrants who arrived in Finland between 2008–2010. For additional analyses on cohort differences, we also included immigrants who have arrived earlier. We restrict the analysis sample to population aged 18–64 and those who are observed in the data for the total 7-years observation period (2011–2017). These criteria leave us with a sample of 296,956 individuals, and 2,078,692 observations in the pooled data 2011–2017. Because all analyses are conducted separately for Finnish-born and immigrant groups, the differing sampling fractions do not bias the within-group estimates. The Finnish subsample is random and therefore representative of that population, while the immigrant group is exhaustive. For this reason, and because our models control for demographic and socioeconomic characteristics (e.g., age, sex, and socioeconomic status) that would typically underlie sampling weights, we do not apply weighting in the analyses.

Of all the immigrants aged 18–64 who arrived in Finland 2008–2010, around 67 per cent remain in Finland in 2017 and are included in the analyses. On average, those who are included in the analytical sample are younger than those who leave Finland before the end of the follow-up and have slightly higher health care costs at the baseline when they come from other Eastern Europe, Asia, Africa and Middle East, but lower costs when they come from Western countries, Russia and Estonia. It is important to note that our results consider a group of immigrants that have become longer-term residents in the country, which makes sense when we are studying health care as an aspect of integration.

### 3.2 Variables

Our outcome variable is health care use and health care costs. The information on health care visits is derived from the Finnish Care Register for Health Care (HILMO), which includes inpatient care from both public and private providers, as well as specialized outpatient care exclusively from public providers. The cost information for psychiatry services is estimated based on the cost information reported by the hospitals, while for other services, the health care classification system (DRG) is used to productizing and pricing. The cost data has been adjusted to reflect the value of money in the year 2020. The complete cost data is available only for years 2011–2017, which limits our observation period to these years. Using costs as the outcome provides a unified metric that captures both the volume and intensity of health care use across different types of services. This measure aggregates expenditures from inpatient and outpatient care, specialist and psychiatric services, and expensive medications consumed in the hospital as part of the treatment. By using total costs rather than simple counts of visits or hospitalizations, we account for the heterogeneity in service intensity, treatment duration, and resource use. In addition to total health care costs, we estimate models with a binary outcome indicating any use of health care services and health care costs among those who have used services. This distinction allows us to separate differences in the likelihood of using any services (extensive margin) from differences in the amount or intensity of care among those who use services (intensive margin), clarifying whether observed cost differences reflect variation in access to care or in the level and type of services utilized.

Our principal independent variable is immigrant status. Immigrants are classified into eight groups by their country of birth as follows: 1) Western countries (including Western Europe together with USA, Canada, Australia and New Zealand), 2) Russia / former USSR, 3) Estonia, 4) other Eastern European countries, 5) Middle East, 6) Asia, 7) Africa, 8) other or unknown (not included in growth curve models). Individuals born in Finland or individuals born abroad to Finnish-born parents are categorised as native-born Finns.

Control variables included in the models are gender, age as a continuous variable, region of residence, labour market status (employed or not), annual taxable income of the individual as a continuous variable (in Euros), and the number of children below the age of 18 in the household. We control for age in every model, as individuals get older over the period, and older people are known to generally use more health care.

These background characteristics are presented in Table 1. There are some differences between immigrant groups and the native Finns. The native-born Finns are on average older, close to 42 years, while immigrants from Middle East and Africa are on average around 33 years old. The Finns and immigrants from Western countries have significantly higher income level, and native Finns and Estonians are more likely to be employed. More than a third of Estonians and Africans were never in a partnership during the observation period, while a majority of Western immigrants was in a partnership with a native-born Finn (67 %). In other immigrant groups, the share was significantly lower.

**Table 1:** Basic statistics (pooled data 2011–2017).

	Number of observations	Share of women	Mean age	Std.dev. age	Mean number of children	Std.dev. number of children	Mean personal income (EUR)	Std.dev. personal income (EUR)	Share employed	Share with non-Finnish partner	Share with Finnish partner	Share no partner
Finnish	1 834 693	49 %	41.7	12.2	0.67	1.05	32 271	21 781	76 %	2 %	62 %	37 %
Western countries	19 516	32 %	36.1	8.5	0.63	0.96	30 100	25 597	56 %	11 %	54 %	35 %
Russia/USSR	37 877	61 %	38.4	10.6	0.71	0.94	18 754	15 562	47 %	53 %	14 %	32 %
Estonia	38 962	51 %	39.5	10.4	0.59	0.93	25 234	16 038	68 %	40 %	7 %	53 %
Other Eastern Europe	22 253	46 %	35.4	8.7	0.79	1.01	22 206	15 506	56 %	51 %	15 %	35 %
Middle East	33 159	30 %	32.8	8.3	0.80	1.14	14 008	12 589	34 %	41 %	12 %	47 %
Asia	47 075	57 %	34.0	8.0	0.59	0.89	18 886	15 499	51 %	35 %	27 %	38 %
Africa	26 509	39 %	32.7	8.3	0.96	1.41	14 838	10 419	39 %	29 %	11 %	60 %
Other/unknown	10 388	42 %	36.3	8.7	0.62	0.90	22 218	16 520	58 %	25 %	34 %	41 %
*All immigrants*	*235 739*	*47 %*	*35.7*	*9.4*	*0.70*	*1.04*	*20 019*	*16 389*	*51 %*	*38 %*	*19 %*	*43 %*

### 3.3 Moderating variables on other aspects of integration

We further study the role of other aspects of integration in health care use. We use different proxies for socio-cultural integration, namely partnership with a native-born Finn, living in a neighbourhood with dominantly native population, and working in a workplace with dominantly native-born co-workers. As for economic integration, we examine the role of employment and income as moderating factors between immigrant status and health care use. It should be noted that these factors – being married in general, being employed, and having higher income – are generally associated with lower health care costs in the native population. Therefore, while they are good proxies for integration, they are also likely to be associated with even lower costs among the immigrants too. Consequently, the comparison group should be native-born Finns in the same sociodemographic or socioeconomic category.

Individuals are categorised into three groups based on partnership status: 1) has a non-Finnish partner (at any point during the observation period), 2) has a Finnish partner (at any point during the observation period), 3) has never been in a partnership during the observation period. If the person is registered to have both a Finnish origin and a non-Finnish origin partner at some point during the observation period, the person is categorised in group 2. Partnerships include both marriages and cohabitation.

Employment status is based on the status at the end of the year and is categorised either as being employed or not. Individuals are grouped into two groups based on equivalised disposable household income: those whose income is below the native-Finn median and those whose income is equal or above the median. Median is calculated from the sample’s native-born Finns separately for each year. Neighbourhood (at a post code level) and workplace characteristics refer to living and working in a place with <75 per cent of native-born Finns.

### 3.4 Methods

Growth curve models are appropriate for modeling trajectories over time and across different groups as they capture initial variation in health care use and trajectory over time within individuals (e.g., Jackson, 2015). In our models, time (*t*) refers to calendar years 2011–2017 when outcomes are measured. We estimate linear models with random intercept and random slope by immigrant group. Preliminary tests comparing linear and quadratic time specifications using likelihood-ratio tests indicated that the linear growth model provided a better fit to the data; therefore, all analyses were conducted using a linear time specification.

In addition, we run separate models by partnership status, employment status, and income level to investigate the role of cultural and economic capital as well as separate models by region of origin. We interpret group differences in health care use trajectories based on average marginal effects derived from the growth curve models. Because the main group coefficient represents only the difference at the baseline year (2011), we rely on predicted values over time to illustrate how trajectories evolve. Marginal predictions were estimated using Stata’s margins command, averaging over the observed covariate distributions. Figures display these predicted values and demonstrate changes in the gap between groups over time. Our assessment of convergence towards the native level relies on looking at the marginal effects in the figures and detecting whether the gap between immigrant groups and natives diminish over time. This takes into account any changes in the background factors that affect health care use.

Our main outcome variable is annual health care costs $y_{it}$ for individual *i* in year *t*. To estimate the linear effect of time, we specify:

(1) $$y_{it}=\;\alpha_{i}+\beta_{i}\left(\;time_{it}\right)+\varepsilon_{it}$$

where $\alpha_{i}$ and $\beta_{i}$ represent individual-specific intercepts and slopes and $\varepsilon_{it}$ is person-year error term. The model allows trajectories to vary as a function of individual covariates, for example:

(2) $$\alpha_{i}=\;\alpha_{0}+\alpha_{1}x_{i1}+\alpha_{2}x_{i2}+\alpha_{k}x_{ik}+\mu_{i}$$

We are especially interested in estimating random slopes by country of origin (and other aspects of integration) as specified in:

(3) $$y_{it}=\;\alpha_{i}+\beta_{i}\left(\;time_{it}\right)+\sum_{c}\gamma_{c}\;country_{ic}+\sum_{c}\delta_{c}\;\left(country_{ic}\;x\;time_{it}\right)+\varepsilon_{it}$$

where $country_{ic}$ are dummies for immigrant groups. $\gamma_{c}$ is the baseline for the group and $\delta_{c}$ gives the difference in slope (or trend over time) relative to the reference group (native Finns).

We use this same model specification to estimate health care use, defined as a binary variable of any costs during the year. The binary outcome is modelled using a linear probability model.

## 4 Results

### 4.1 Descriptive evidence on costs by category

Based on Table 2 (averages by cost category), we see that native Finns have annual total costs of around 925 Euros, around double compared to immigrants from Western countries or Asia. Costs among immigrants from Middle East and Africa are close to the Finnish average. We also see remarkable differences across cost categories: it is especially psychiatric costs that are higher among the native Finns. In the following, we only focus on total health care costs or use.

**Table 2:** Average annual costs (and standard deviations) in euros by service categories (pooled data 2011–2017).

	All costs, mean	All costs, sdt.dev.	Psychiatric costs, mean	Psychiatric costs, std.dev.	Somatic costs, mean	Somatic costs, std.dev.	Fertility-related costs, mean	Fertility-related costs, std.dev.	In-patient costs, mean	In-patient costs, std.dev.	Out-patient costs, mean	Out-patient costs, std.dev.	ER costs, mean	ER costs, std.dev.
Finnish	925	4818	203	3248	722	3447	79	725	453	4132	472	1803	62	279
Western countries	450	2037	74	1095	376	1644	86	583	181	1416	269	1036	40	162
Russia/USSR	515	2688	33	888	481	2515	154	825	248	2153	267	1128	46	173
Estonia	525	2620	55	1149	470	2294	88	562	254	2146	271	966	48	191
Other Eastern Europe	549	2240	38	683	511	2106	158	823	241	1558	308	1190	62	219
Middle East	847	5048	129	2785	718	4112	155	879	428	4547	419	1442	91	266
Asia	454	2307	34	781	420	2149	165	837	240	1945	214	832	40	164
Africa	884	3913	95	2101	788	3273	279	1251	469	3233	414	1612	85	244
Other/unknown	517	1831	57	712	459	1638	128	815	211	1308	305	937	58	213
All immigrants	590	3107	62	1490	528	2678	153	843	290	2612	301	1161	57	205

### 4.2 Results based on growth curve models

**Fig. 2:**
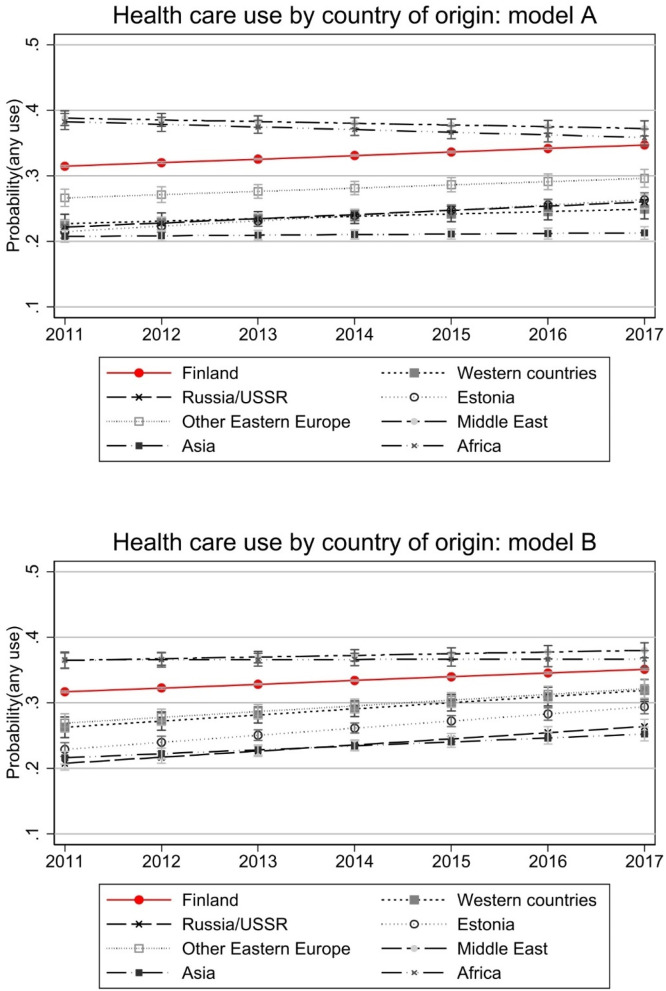
Health care use by country of origin. Note: Model A controls for age and gender. Model B controls for age, gender, region of residence, employment, annual taxable income, and number of children in the household. Y-axis denotes the probability of having any health care costs, i.e., use of health care. Full models are presented in Appendix Table A1.

**Fig. 3:**
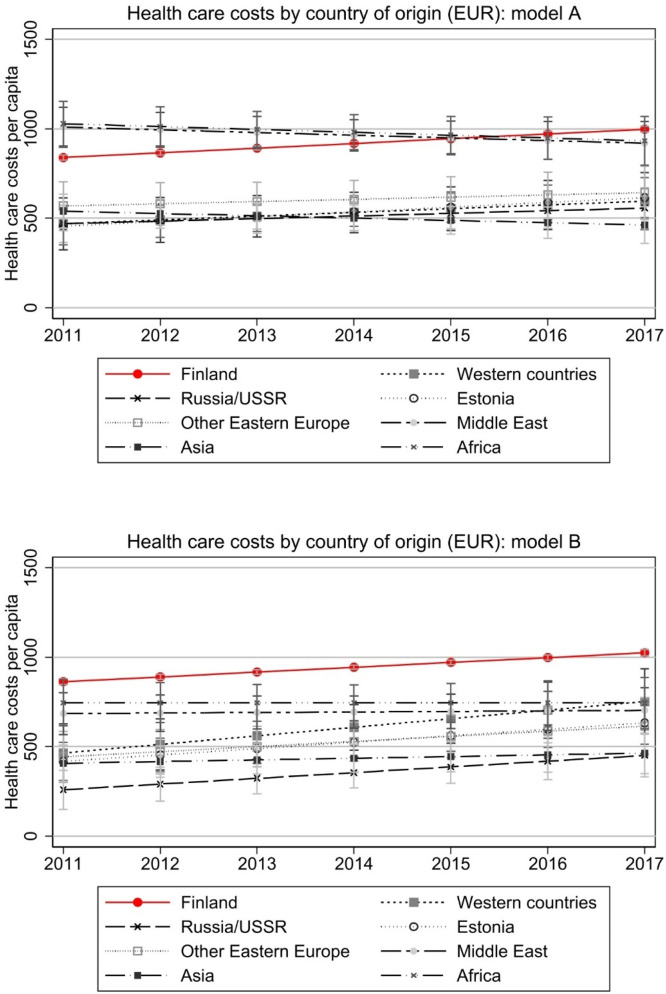
Total health care costs by country of origin. Note: Model A controls for age and gender. Model B controls for age, gender, region of residence, employment, annual taxable income, and number of children in the household. Costs are deflated to the price level of 2020. Full models are presented in Appendix Table A1.

We move on to results where we control for important background factors associated with health care use. Fig. 2 illustrates probability for using any specialised health care during the year controlling for age and gender (left panel) and additionally controlling for employment, personal annual income, number of children in the household, and region of residence. Fig. 3 illustrates similarly the estimated annual total costs in Euros. There appears to be modest convergence when overall use is the outcome, while no convergence in costs. Adding more control variables in the model affects the group trajectories or ordering of countries, but not convergence. The native-immigrant difference appears statistically significant in almost all immigrant groups and the confidence intervals are narrow in all groups.

**Fig. 4:**
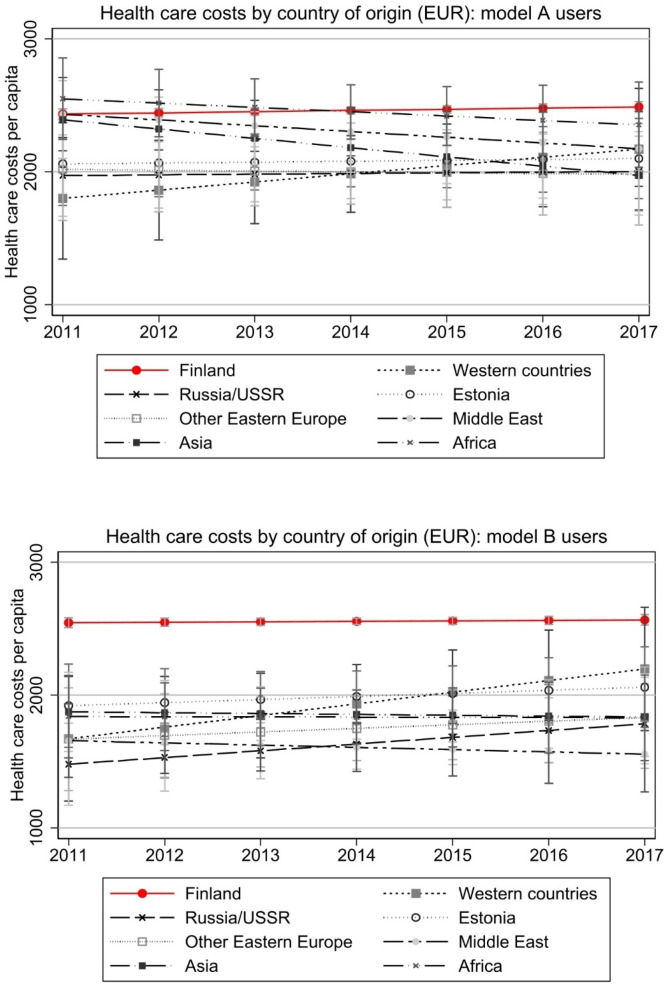
Total health care costs of those with any use. Note: Model A controls for age and gender. Model B controls for age, gender, region of residence, employment, annual taxable income, and number of children in the household. Costs are deflated to the price level of 2020. Full models are presented in Appendix Table A2.

The differences between immigrants arriving in Finland between years 2008 and 2010 and natives are large in 2011 and remain so through the follow-up period, with most immigrant groups using significantly less health care. Interestingly, immigrants from Africa and Middle East seem to have used more health care in 2011 than natives, but their average health care use drops over the years. Their higher costs are partly explained by background characteristics as this is not visible in model B of Fig. 3. Also, while their probability of use remains slightly higher than among natives (Fig. 2), their costs in 2017 are significantly below that of natives (Fig. 3). If there is any convergence in costs, it regards to immigrants from Western countries with steeper than average increase in both use and costs. In Fig. 4, we further look at costs among those with any use. This shows that native-immigrant gap is considerable also among those who use services (with wider and partly overlapping confidence intervals in Model A), and convergence mainly happens between immigrants from Western countries and Estonia and native Finns.

**Fig. 5:**
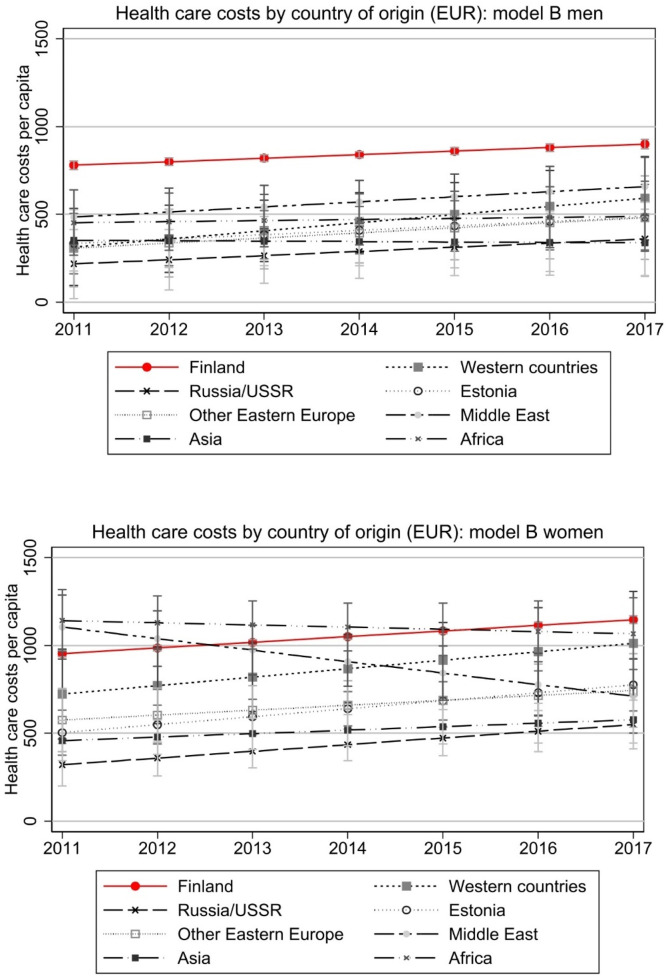
Total health care costs by country of origin, separately for men and women. Note: Model controls for age, region of residence, employment, annual taxable income, and number of children in the household (as in Model B in Fig. 2). Costs are deflated to the price level of 2020. Full models are presented in Appendix Table A2.

Fig. 5 additionally shows results of model B separately for men and women. While the results regarding men follow the overall pattern in Fig. 3, among women there are more diverse patterns to be seen (however, with wider and partly overlapping confidence intervals): convergence in costs among Western immigrants, but divergence regarding women from Middle East. As in the native population, the costs among women are higher than among men.

**Fig. 6:**
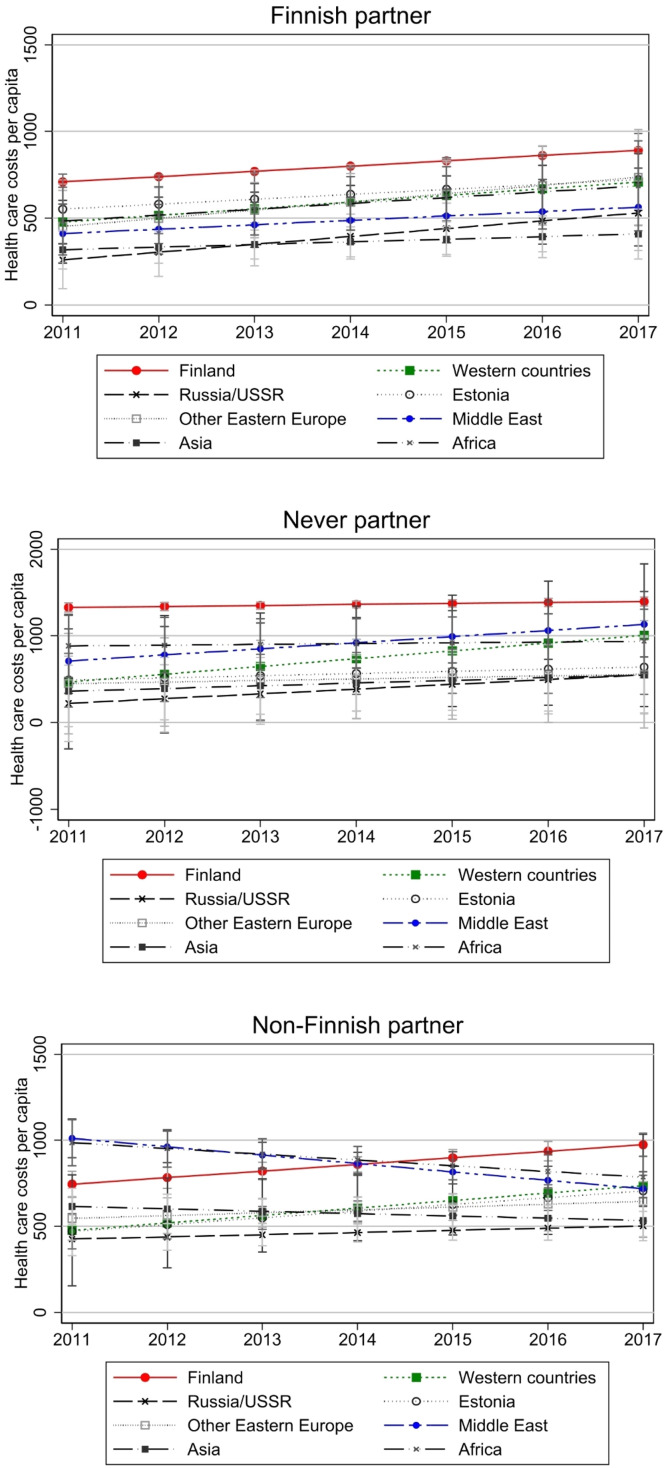
Health care costs by partnership status. Note: Model controls for age, gender, region of residence, employment, annual taxable income, and number of children in the household. Models have been run separately by partnership status. Costs are deflated to the price level of 2020. Full models are presented in Appendix Table A3. Note that y-axis scale is different for the group “never partner”.

Fig. 6 graphs health care costs by partnership status. In Appendix Fig. A1, graphs can also be found in separate plots by country of origin to compare the role of partnership status within groups. For natives, health care use is higher for those with no partner. The health premium of marriage (or health selection into marriage) is well documented in the literature (e.g., Dupre et al., 2009; Lawrence et al., 2019), and these results seem to confirm this for health care costs as well. The results differ between the immigrant groups and are not easily summed. Also, overlapping confidence intervals illustrate that not all groups are statistically different from natives. Whether the partner is Finnish or non-Finnish does not seem to matter in all groups, and among Estonian, Russian, and other Eastern European immigrants, those with a Finnish partner, have the highest costs and not those without a partner like in other groups at the end of the observation period. For Africans, we see health care use decreasing for those with non-Finnish partner, while it increases slightly for those with Finnish partner. What is curious is that for Western and Middle East immigrants, health care use grows strongly over the period for those with no partner, compared to those with partner. These results might reflect selection into partnership with natives or in general as discussed by Vaalavuo and Rask (2024). Therefore, rather than being a proxy of integration, a Finnish partner (or any partner, for that matter) might reflect health selection into partnership, which is stronger among some groups than others. All in all, there seems to be no convergence in costs when partner is a native Finn, against our hypothesis.

**Fig. 7:**
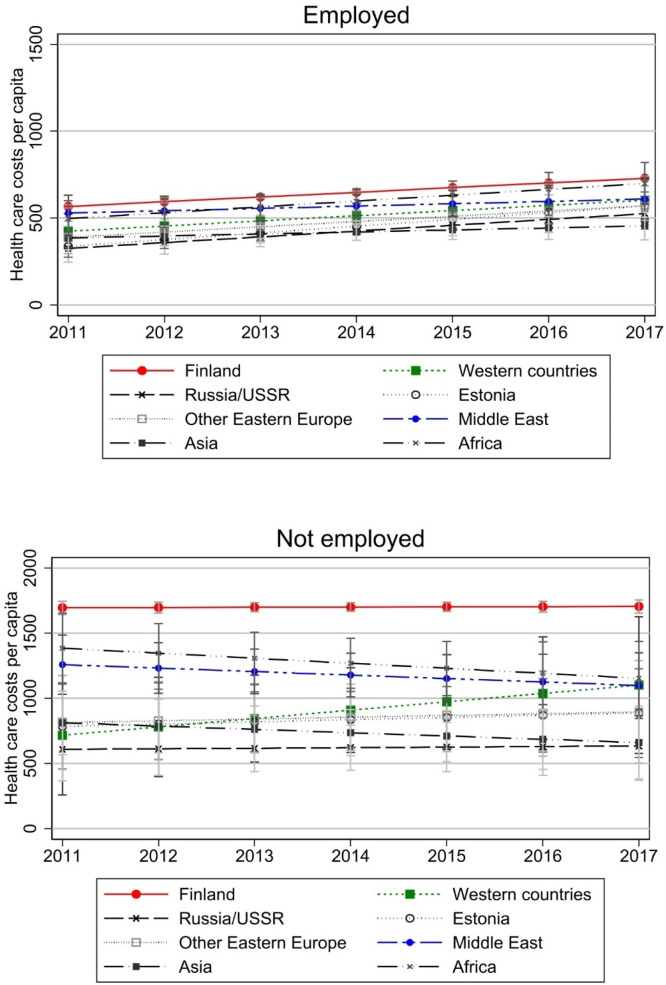
Health care costs by employment status. Note: Model controls for age, gender, region of residence, annual taxable income, and number of children in the household. Models have been run separately by employment status. Costs are deflated to the price level of 2020. Full models are presented in Appendix Table A4. Note that y-axis scales are different.

In Fig. 7, employed and non-employed are compared. In Appendix Fig. A2, the results are plotted by country. In these figures we see a more systematic pattern that in every group non-employed have higher costs than employed. However, as with native-Finns, the costs of employed seem to grow slightly more over time in most groups, especially among African immigrants, while the opposite is true for Western immigrants. Foreign-born employed are generally closer to the cost level of the natives and there are only modest differences in costs. This would partly support our expectation that integration in one area is associated with smaller native-immigrant gap in health care costs.

**Fig. 8:**
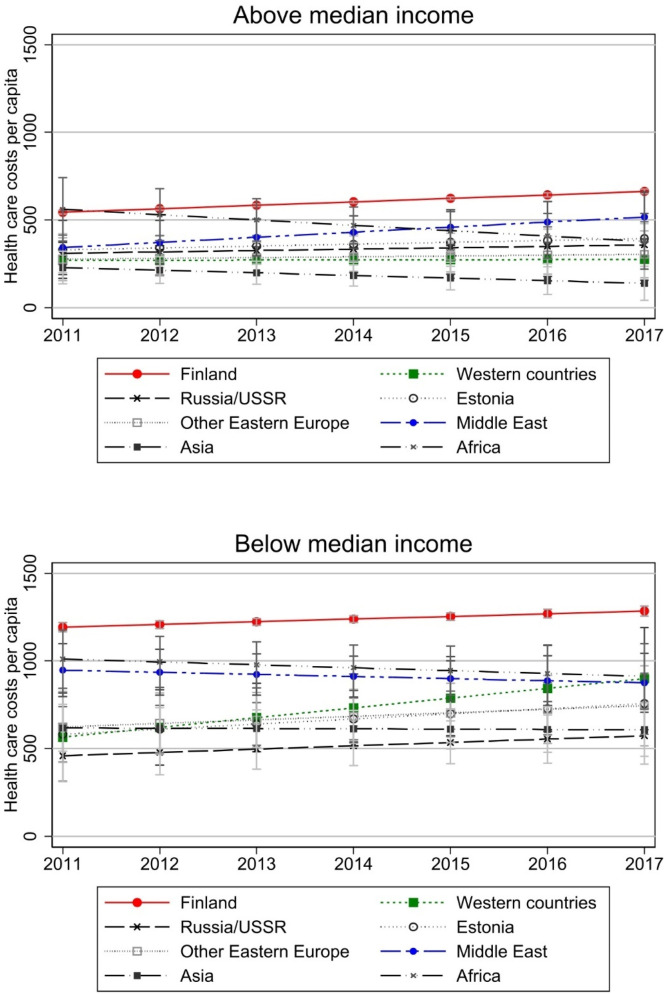
Total health care costs by relative income level, separately by country of origin. Note: Relative income status refers to income lower than the median of native-born Finns in the sample and the year in question and equal or higher than the median. model controls for age, gender, region of residence, employment, and number of children in the household. models have been run separately by income status. Costs are deflated to the price level of 2020. Full models are presented in Appendix Table A5.

Similarly, results by relative income level are presented in Fig. 8 and separately by country in Appendix Fig. A4. Again, the common pattern is that those with lower income (below median income) have higher costs. Fig. 8 also illustrates that there seems to be rather divergence than convergence in costs when those with income above the median are analysed. However, as with employment, the native-immigrant gaps in costs are smaller among those having above median income. In other words, if we do not consider convergence over time, we might also conclude that economic integration is definitely associated with smaller gaps in health care use.

In Table 3 only immigrants are included in the analyses because very few native Finns work or live in places with <75 per cent of native-born Finns (see Appendix Table A6). In addition, the workplace information is naturally only available for employed immigrants. For comparison, we also include results on neighbourhood characteristics for those employed. The results show that neighbourhood characteristics – as we have defined them – are not statistically significantly associated with health care costs. However, working in a workplace with a lower native share seems to be associated with lower costs supporting our expectation. However, it is also possible that this workplace characteristic is associated with unobserved characteristics of immigrants, a result of non-random selection into such workplaces.

**Table 3:** Results from growth curve models with neighborhood and workplace characteristics.

	Workplace, only employed immigrants	Neighborhood, all immigrants	Neighborhood, only employed immigrants
Time	26.122*	35.759^⁎⁎^	23.623*
	(11.054)	(13.686)	(11.988)
Immigrant group (ref. Western immigrants)			
Russia/USSR	−76.637	−160.105*	−85.828
	(59.218)	(77.110)	(68.691)
Estonia	−61.923	−75.850	−77.372
	(54.513)	(76.525)	(63.608)
Other Eastern Europe	−25.786	−16.165	−46.193
	(62.476)	(84.584)	(72.838)
Middle East	154.390*	394.680^⁎⁎⁎^	167.062*
	(67.160)	(78.612)	(77.447)
Asia	−33.413	−9.836	−6.373
	(55.790)	(75.089)	(64.769)
Africa	124.295	421.205^⁎⁎⁎^	115.177
	(66.423)	(81.664)	(77.490)
Other/unknown	15.128	−29.793	12.759
	(75.917)	(103.144)	(88.533)
Age	1.448	3.609^⁎⁎^	1.622
	(0.898)	(1.103)	(0.957)
Female	321.801^⁎⁎⁎^	410.762^⁎⁎⁎^	337.159^⁎⁎⁎^
	(16.299)	(20.729)	(17.232)
Number of children	89.852^⁎⁎⁎^	117.931^⁎⁎⁎^	96.134^⁎⁎⁎^
	(8.221)	(8.599)	(8.579)
Personal annual income	−0.001*	−0.001	−0.000
	(0.000)	(0.001)	(0.000)
Native share at workplace <75 %	−49.908^⁎⁎⁎^		
	(14.750)		
Employed		−237.345^⁎⁎⁎^	
		(18.221)	
Native share in neighborhood <75 %		14.154	10.224
		(31.599)	(30.492)
Constant	129.138*	220.338^⁎⁎^	78.338
	(56.566)	(75.421)	(63.522)
*Observations*	*115,972*	*202,885*	*116,055*

Note: Region of residence and interaction between time and country of origin are also controlled for, but not shown, as we are mainly interested in the coefficient for “native share”. Standard errors in parentheses, * *p* < 0.05, ^⁎⁎^*p* < 0.01, ^⁎⁎⁎^*p* < 0.001.

**Fig. 9:**
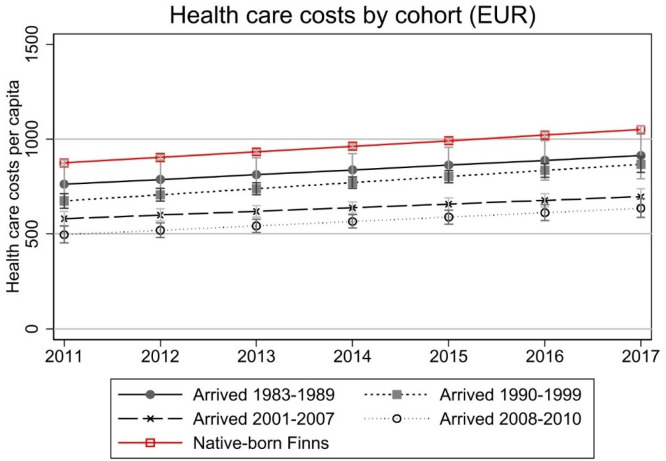
Health care costs by immigrant cohort. Note: Model controls for age, gender, region of residence, employment, personal taxable income, and number of children in the household. costs are deflated to the price level of 2020. full models are presented in Appendix Table A6. As country of origin is not known for all immigrants who have arrived in the 1980s, we do not control it in the model.

Finally, we extend the analysis to immigrant cohorts that have arrived in Finland since the 1980s. This is to see whether a much longer residence in Finland affects the results and whether we can (finally) observe patterns of convergence in costs. This indeed seems to be the case as earlier arrived immigrants have higher costs than more recent immigrant cohorts (Fig. 9). We can observe that each subsequent cohort has smaller costs. However, it should be highlighted that immigrant cohorts that have arrived in the early 1980s differ from the most recent cohorts in many ways, also in terms of country of origin, age at migration, and reason for migration. Also, we do not observe their health care costs immediately after arrival, so we do not know whether they have started with similar health costs relative to natives as the cohort of 2008–2010 immigrants under focus in the above analyses.

## 5 Conclusions

In this article we examined the health care use and costs among a cohort of recently arrived working-age immigrants in Finland and studied whether their costs converge to the native levels over a 7-year observation period. The study contributes to the literature on the healthy immigrant effect, ethnic health and service-use disparities, and implications of rising immigration for national and regional health care costs. We approached the convergence of health care costs between immigrants and natives as a potential indicator of integration within the health care system, expecting to see convergence in costs over time and especially along other dimensions of integration. We studied both proxies for cultural and social integration as well as economic integration. This evidence in a novel contribution to the prior literature.

The results are important as differences in population structure affect regional health disparities and the need for health care services. Immigration is likely to increasingly affect the population structure in Finland, and therefore also the health care needs across regions. Immigrants are on average younger than native population and tend to live in urban areas. However, immigrants too get older and need more health care as they age. While the *healthy immigrant thesis* expects lower costs and use among immigrants, the *acculturation thesis* expects the health status of immigrants to converge to native level over time. The acculturation thesis attributes immigrants’ initial health advantage to healthier life styles (Hagos and Hamilton, 2024). However, we argue that lower initial costs may also reflect unmet needs arising from, for example, limited knowledge about available services. Therefore, integration in other areas of life would promote more equal use of services and thereby convergence in costs over time. As we note, there might be health-related selection in other dimensions of integration, for example in being employed or having a native partner, therefore we need to be prudent in drawing causal inferences and rather illustrate the various interrelationships between these factors.

We found that health care costs are lower among immigrants compared to natives even (or especially) when controlling for various background characteristics. There also appears to be almost no convergence over time. However, differences between immigrant groups and cost categories are clearly visible, but quite systematically only immigrants from Western countries seem to slightly approach the cost level of native Finns. We also noted that other dimensions of immigrant integration (having a native partner, neighborhood and workplace characteristics, and socioeconomic status) have some role in moderating the association between immigrant background and health care costs.

The native-immigrant cost gap was smaller and sometimes differences were not statistically significant when we analyzed different groups that can be considered more culturally or economically integrated. Moreover, these factors – such as having a partner and higher income – appeared to affect costs the same way among natives and immigrants in most cases. The smallest gap in costs appeared among employed immigrants and natives. Therefore, economic integration was indeed strongly associated with smaller differences in costs between any immigrant group and natives. The results could also indicate that there is positive health selection to having higher income, being employed, and having a Finnish partner. Such non-random selection regarding unobserved characteristics makes it difficult to analyze the connection between integration and health service use, but the results might suggest which groups should be targeted to improve access to health care. We found remarkable variation in health care costs when looking beyond the averages and even within immigrant groups from same regions. The variation in the results demonstrate that the healthy immigrant effect is an oversimplification of the reality. On the other hand, our results together with the results found by Kemppainen et al. (2024) concerning lower mortality risk for older migrants, could indicate that migrants tend to be healthier on average and that convergence may take more time as suggested by the results on earlier immigrant cohorts.

While the analyses are based on rich register data, we were not able to account for all factors affecting health care need. Therefore, there is causal ambiguity in the findings, and we are currently not able to say whether the findings indicate unequal access, unmet needs, efficiency of care or better health status among most immigrant groups. This also makes it challenging to interpret potential convergence as integration, and other data sources, including survey data, might be necessary to complete the analysis of health care use as an aspect of immigrant integration. Future research should link administrative cost data with diagnostic, morbidity, and mortality data, as well as survey data on perceived needs, barrier in access and perceptions of quality of services to clarify whether observed cost differences stem from differences in health needs, access, or efficiency of care. Previous evidence suggests that immigrants face considerable obstacles in accessing health care and suffer from unmet health care needs also in Finland (Kuusio et al., 2023). However, when we look at immigrants who have a Finnish partner, we would assume that they would get the relevant information on accessing the services they need, which might indicate that at least those immigrants with a native partner might indeed be in better health than the natives. Further studies should explore whether the gender of the Finnish partner may have an influence, as it may be that native Finnish women are more informed of the Finnish health care system than native Finnish men. Additionally, further studies should explore the sector of employment and reasons for migration, as it is possible that the persons in this group are highly skilled international migrants and may be therefore more likely to be selected with better health.

The finding on the cost differences among service users could also indicate that immigrants do not need as intensive and expensive care as the native Finns. However, this result could also indicate that even when immigrants access services, they do not get equal care compared with natives. Culturally competent health systems should be developed in increasingly diverse societies, while effectiveness of tools to this end should be improved (Anderson et al., 2003). These are questions that need to be tackled in future research with a more focused research design suitable for disentangling such questions. Despite these limitations, the use of register data is promising and has many advantages. For example, prior evidence has demonstrated that immigrants report more health care visits in surveys than is observable in administrative data (García-Velázquez et al., 2022). Disparity between register and self-reported data is likely to be at least partially attributable to regional differences in how complete the administrative health register data is in some regions. Further studies should consider using multisource data to get a more complete overview of the use of services, and background factors associated with service use. The current study used data up to 2017, which is the time prior to Covid-19 pandemic. Further studies should explore whether the need for care and health care costs have changed after the pandemic.

In the future research on the topic, it would be important to analyze outcomes of health care use in different immigrant groups, and the social and economic consequences of ill health among immigrants. Some evidence already suggests that outcomes are worse among immigrants, for example cancer mortality among immigrant children is significantly higher than among native children (Kyrönlahti et al., 2020). Also, as was suggested by descriptive evidence of our data, a considerable difference in costs exists across health care sectors – a topic that should be investigated further. A few concrete implications for health care providers could be highlighted. First, regional service providers should disaggregate data on service use by region of origin in order to identify groups with high and low health care use, and to determine the underlying mechanisms. Lower use of health care does not necessarily equate to better health. Second, if lower health care use is found to be related to unmet health needs, emphasis should be placed on inclusive and accessible communication, culturally appropriate care, and addressing implicit bias among the health care professionals.

## CRediT authorship contribution statement

**Maria Vaalavuo:** Writing – original draft, Supervision, Project administration, Methodology, Funding acquisition, Formal analysis, Data curation, Conceptualization. **Tuukka Holster:** Writing – original draft, Conceptualization. **Natalia Skogberg:** Writing – original draft, Conceptualization. **Heidi Kuusinen:** Writing – original draft, Data curation.

## Declaration of competing interest

The authors declare that they have no known competing financial interests or personal relationships that could have appeared to influence the work reported in this paper.

## Appendix Supplementary materials

Image, application 1

## Footnotes

1 For more information, see https://invest.utu.fi/fi/investkokonaisdata/information-about-the-research/

## References

[bib0001] Aaltonen K., Vaalavuo M. (2024). Financial burden of medicines in five Northern European countries: a decommodification perspective. Soc. Sci. Med..

[bib0002] Akhtar S.S., Heydon S., Norris P. (2022). Access to healthcare system: experiences and perspectives of Pakistani immigrant mothers in New Zealand. J. Migr. Health.

[bib0003] Andersen R., Newman J.F. (1973). Societal and individual determinants of medical care utilization in the United States. Milbank Meml. Fund. Q Health Soc..

[bib0004] Anderson L.M., Scrimshaw S.C., Fullilove M.T., Fielding J.E., Normand J. (2003). Culturally competent healthcare systems: a systematic review. Am. J. Prev. Med..

[bib0005] Arsenijevic J., Seibel V. (2024). Do immigrants know less than natives about cancer screening tests? The case of Netherlands. J. Migr. Health.

[bib0006] Barlow P., Mohan G., Nolan A. (2022). Utilisation of healthcare by immigrant adults relative to the host population: evidence from Ireland. J. Migr. Health.

[bib0008] Çilenti K., Rask S., Elovainio M., Lilja E., Kuusio H., Koskinen S., Koponen P., Castaneda A.E. (2021). Use of health services and unmet need among adults of Russian, Somali and Kurdish origin in Finland. Int. J. Env. Res. Public Health.

[bib0009] Dehkordy S.F., Hall K.S., Dalton V.K., Carlos R.C. (2016). The link between everyday discrimination, healthcare utilization, and health status among a national sample of women. J. Women's Health.

[bib0010] Domínguez-Rodríguez A., González-Rábago Y. (2024). Self-rated health, time of residence and social determinants of health in immigrant populations: a complex relationship in groups of differentorigins in Southern European region. J. Migr. Health.

[bib0011] Dupre M.E., Beck A.N., Meadows S.O. (2009). Marital trajectories and mortality among US adults. Am. J. Epidemiol..

[bib0012] Elshahat S., Moffat T., Newbold K.B. (2022). Understanding the healthy immigrant effect in the context of mental health challenges: a systematic critical review. J. Immigr. Minor. Health.

[bib0013] García-Velázquez R., Kieseppä V., Lilja E., Koponen P., Skogberg N., Kuusio H. (2022). A multisource approach to health care use: concordance between register and self-reported physician visits in the foreign-born population in Finland. BMC. Med. Res. Methodol..

[bib0014] Gazard B., Chui Z., Harber-Aschan L., MacCrimmon S., Bakolis I., Rimes K., Hotopf M., Hatch S.L. (2018). Barrier or stressor? The role of discrimination experiences in health service use. BMC. Public Health.

[bib0015] Hagos R.M., Hamilton T.G. (2024). Beyond acculturation: health and immigrants' social integration in the United States. J. Health Soc. Behav..

[bib0016] Helgesson M., Johansson B., Nordquist T., Vingård E., Svartengren M. (2019). Healthy migrant effect in the Swedish context: a register-based, longitudinal cohort study. BMJ Open..

[bib0019] Holz M. (2022). Health inequalities in Germany: differences in the ‘healthy migrant effect’ of European, non-European and internal migrants. J. Ethn. Migr. Stud..

[bib0020] Huijts T., Eikemo T.A., Skalická V. (2010). Income-related health inequalities in the Nordic countries: examining the role of education, occupational class, and age. Soc. Sci. Med..

[bib0021] Ichou M., Wallace M. (2019). The Healthy Immigrant Effect: the role of educational selectivity in the good health of migrants. Demogr. Res..

[bib0022] Iglesias E., Robertson E., Johansson S.-E., Engfeldt P., Sundquist J. (2003). Women, international migration and self-reported health. A population-based study of women of reproductive age. Soc. Sci. Med..

[bib0023] Jackson M.I. (2015). Cumulative inequality in child health and academic achievement. J. Health Soc. Behav..

[bib0024] Kemppainen L., Kemppainen T., Raitanen J., Aaltonen M., Forma L., Kouvonen A., Pulkki J. (2024). All-cause and cause-specific mortality among older migrant and non-migrant adults in Finland: a register study on all deaths, 2002–2020. Eur. J. Public Health.

[bib0025] Kennedy S., Kidd M.P., McDonald J.T., Biddle N. (2015). The healthy Immigrant Effect: patterns and evidence from four countries. J. Int. Migr. Integr..

[bib0026] Kieseppä V., Torniainen-Holm M., Jokela M., Suvisaari J., Gissler M., Markkula N., Lehti V. (2020). Immigrants’ mental health service use compared to that of native Finns: a register study. Soc. Psychiatry Psychiatr. Epidemiol..

[bib0027] Kuusio H., García Velázquez R., Mäkipää L., Klemettilä K., Castaneda A., Lilja E. (2023).

[bib0028] Kyrönlahti A., Madanat-Harjuoja L., Pitkäniemi J., Rantanen M., Malila N., Taskinen M. (2020). Childhood cancer mortality and survival in immigrants: a population-based registry study in Finland. Int. J. Cancer.

[bib0029] Laatikainen T., Skogberg N., Koponen P., Koskinen S., Kuusio H. (2020). Ulkomaalaistaustaisten Terveys Ja Hyvinvointi Suomessa. FinMonik-tutkimus 2018-2019.

[bib0030] Lawrence E.M., Rogers R.G., Zajacova A. (2019). Marital happiness, Marital status, health, and longevity. J. Happiness. Stud..

[bib0031] Loi S., Li P., Myrskylä M. (2025). Unequal weathering: how immigrants health advantage vanishes over the life course. J. Migr. Health.

[bib0032] McDonald J.T., Kennedy S. (2004). Insights into the 'healthy immigrant effect': health status and health service use of immigrants to Canada. Soc. Sci. Med..

[bib0033] Pandey M., Kamrul R., Michaels C.R., McCarron M. (2022). Perceptions of mental health and utilization of mental health services among new immigrants in Canada: a qualitative study. Community Ment. Health J..

[bib0034] Rask S., Suvisaari J., Koskinen S., Koponen P., Mölsä M., Lehtisalo R., Schubert C., Pakaslahti A., Castaneda A.E. (2016). The ethnic gap in mental health: a population-based study of Russian, Somali and Kurdish origin migrants in Finland. Scand. J. Public Health.

[bib0035] Rivenbark J.G., Ichou M. (2020). Discrimination in healthcare as a barrier to care: experiences of socially disadvantaged populations in France from a nationally representative survey. BMC. Public Health.

[bib0036] Robertsson T., Kokko S., Lilja E., Castañeda A.E. (2023). Prevalence and risk factors of psychological distress among foreign-born population in Finland: a population-based survey comparing nine regions of origin. Scand. J. Public Health.

[bib0037] Skogberg N., Laatikainen T., Koskinen S., Vartiainen E., Jula A., Leiviskä J., Härkänen T., Koponen P. (2016). Cardiovascular risk factors among Russian, Somali and Kurdish migrants in comparison with the general Finnish population. Eur. J. Public Health.

[bib0038] Skogberg N., Laatikainen T., Lundqvist A., Lilja E., Härkänen T., Koponen P. (2018). Which anthropometric measures best indicate type 2 diabetes among Russian, Somali and Kurdish origin migrants in Finland? a cross-sectional study. BMJ Open..

[bib0039] Solé-Auró A., Crimmins E.M. (2008). Health of immigrants in European countries. Int. Migr. Rev..

[bib0040] Statistics Finland (2023) Online data base of Statistics Finland, data on population structure by country of birth: https://pxdata.stat.fi/PxWeb/pxweb/fi/StatFin/(accessed 18 December 2024).

[bib0041] Straiton M.L., Myhre S. (2017). Learning to navigate the healthcare system in a new country: a qualitative study. Scand. J. Prim. Health Care.

[bib0043] Vaalavuo M., Rask S. (2024). Earnings and employment trajectories among a cohort of new immigrants in Finland: does a native-born partner enhance economic integration?. Eur. Sociol. Rev..

[bib0042] Vaalavuo M. (2020). Use of public health and social care services among the elderly in Finland: an under-examined mechanism of redistribution. J. Eur. Soc. Policy..

[bib0044] van Doorslaer E., Wagstaff A., Bleichrodt H., Calonge S., Gerdtham U.G., Gerfin M., Geurts J., Gross L., Häkkinen U., Leu R.E., O'Donell O., Propper C., Puffer F., Rodríguez M., Sundberg G., Winkelhake O. (1997). Income-related inequalities in health: some international comparisons. J. Health Econ..

[bib0045] Vang Z.M., Sigouin J., Flenon A., Gagnon A. (2016). Are immigrants healthier than native-born Canadians? A systematic review of the healthy immigrant effect in Canada. Ethn. Health.

[bib0046] Willis K., Collyer F., Lewis S., Gabe J., Flaherty I., Calnan M. (2016). Knowledge matters: producing and using knowledge to navigate healthcare systems. Health Sociol. Rev..

[bib0047] Wilson F.A., Zallman L., Pagán J.A., Ortega A.N., Wang Y., Tatar M., Stimpson J.P. (2020). Comparison of use of health care services and spending for unauthorized immigrants vs authorized immigrants or US citizens using a machine learning model. JAMa Netw. Open..

[bib0048] Xu J., Chen X., Liu K., Guo G., Li Y. (2021). Health service utilization of international immigrants in Yiwu, China: implication for health policy. J. Immigr. Minor. Health.

