# Supplementary material for: Health care use as an aspect of immigrant integration? An analysis of health care cost convergence among new immigrants and natives in Finland
Source: J Migr Health. 2025 Dec 11;13:100386. doi: 10.1016/j.jmh.2025.100386 (PMC12771491; doi:10.1016/j.jmh.2025.100386)
Supplement: Supplementary file 1 [file mmc1.docx]

**Appendix Figures and Tables.**

**Appendix Figure A1. Total health care costs by partnership type, separately by country of origin.**


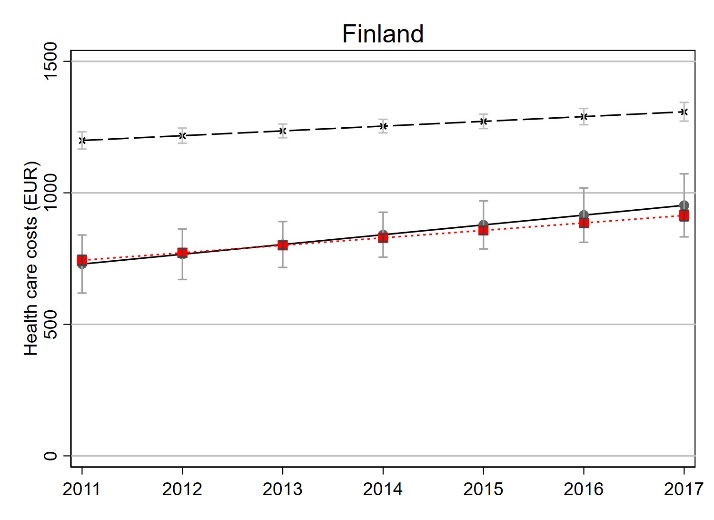

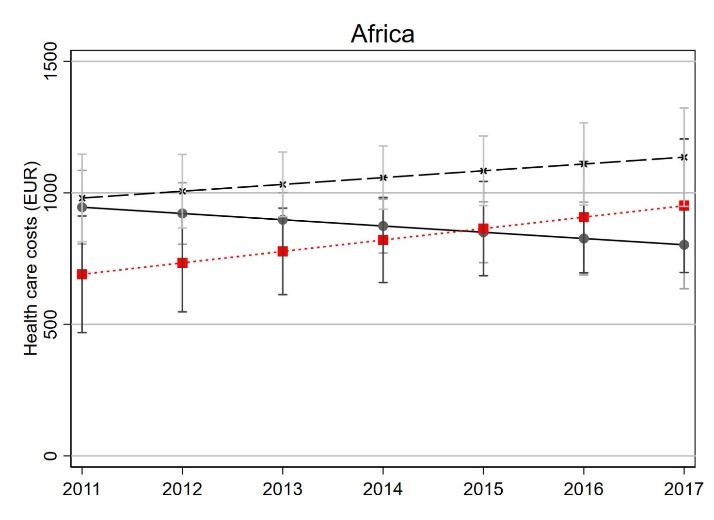


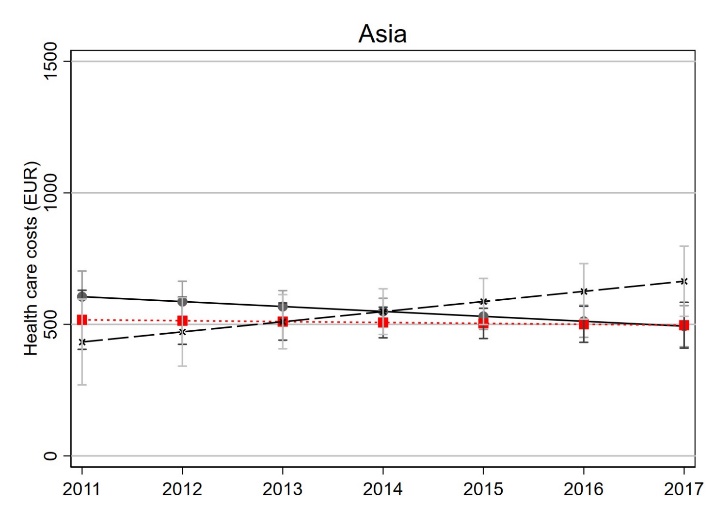

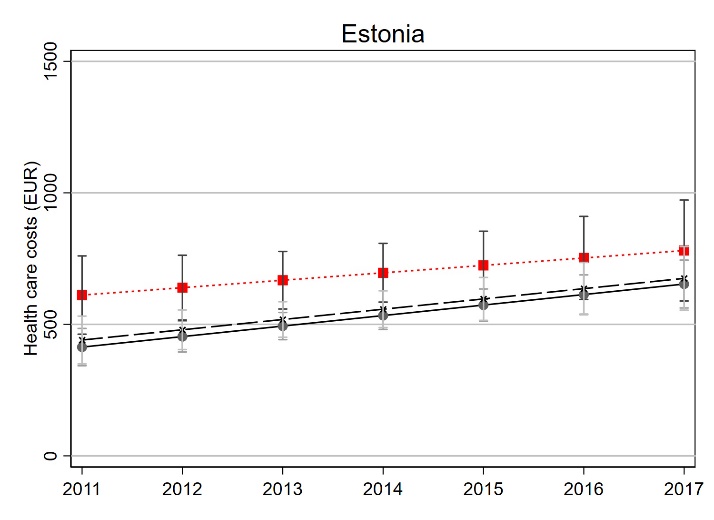


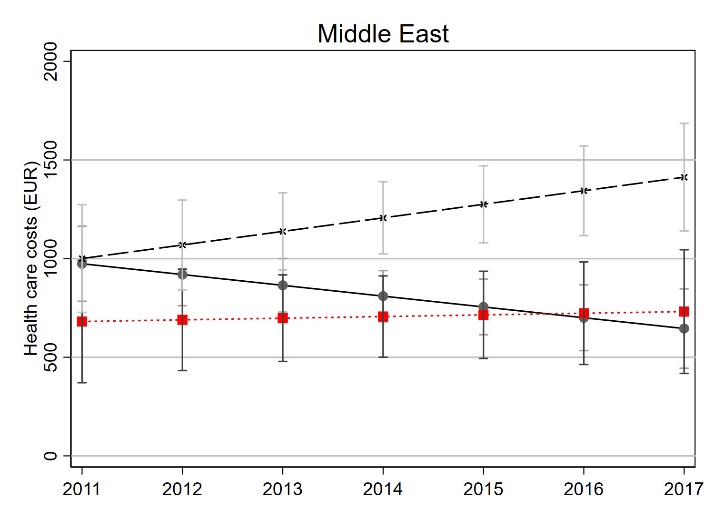

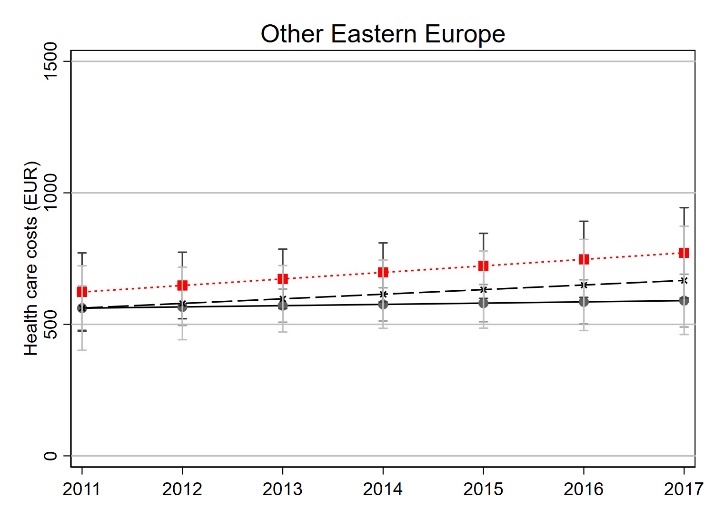


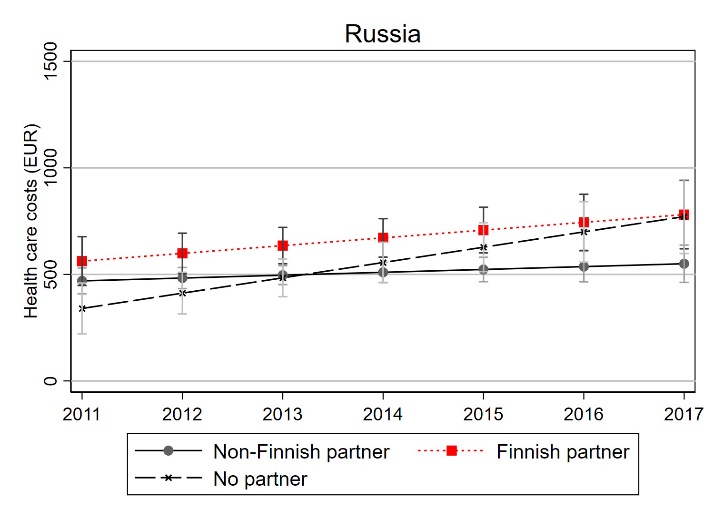

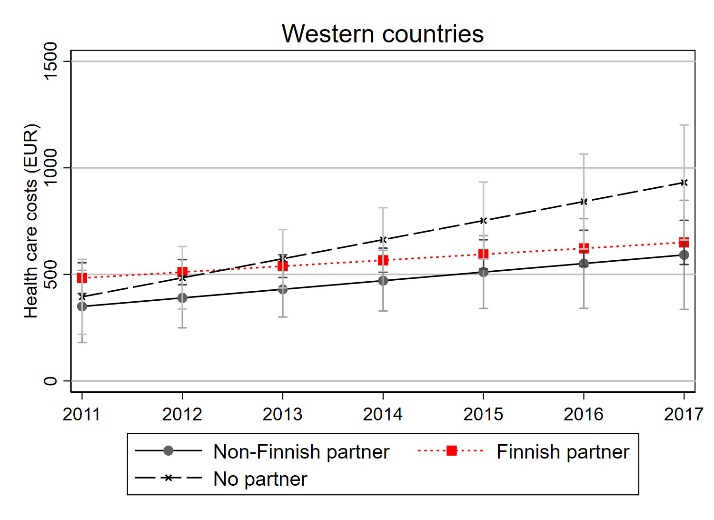


Note: Model controls for age, gender, region of residence, employment, annual taxable income, and number of children in the household. Costs are deflated to the price level of 2020. Models are run separately by country. Note that the Y-axis scale differs for Middle East.

**Appendix Figure A2. Total health care costs by employment status, separately by country of origin.**


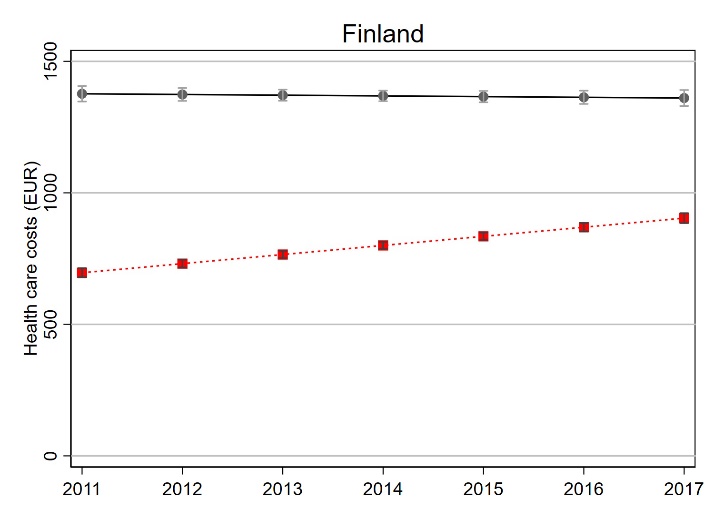

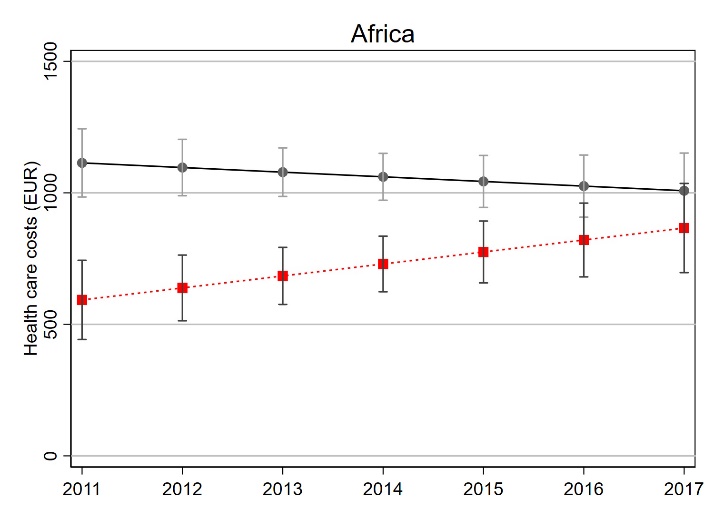


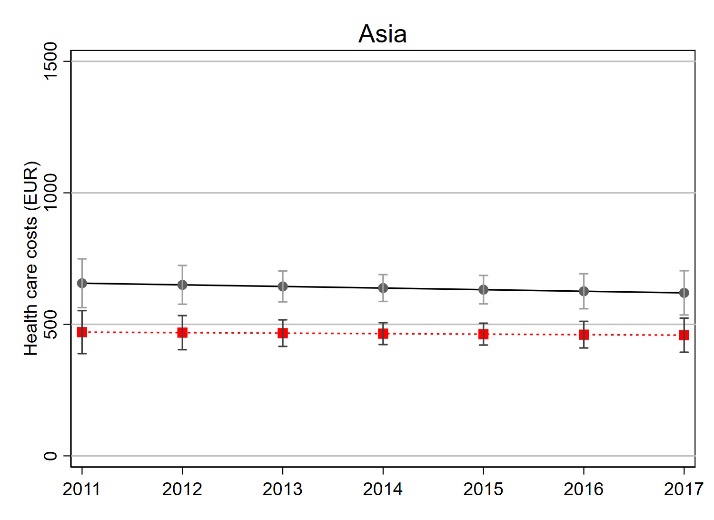

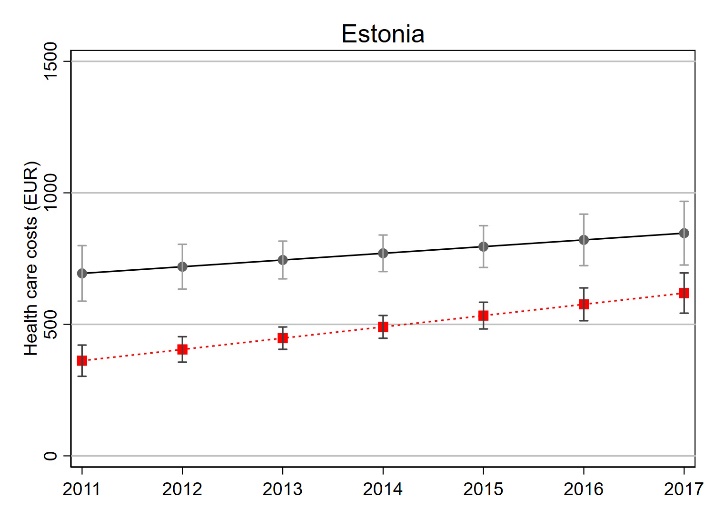


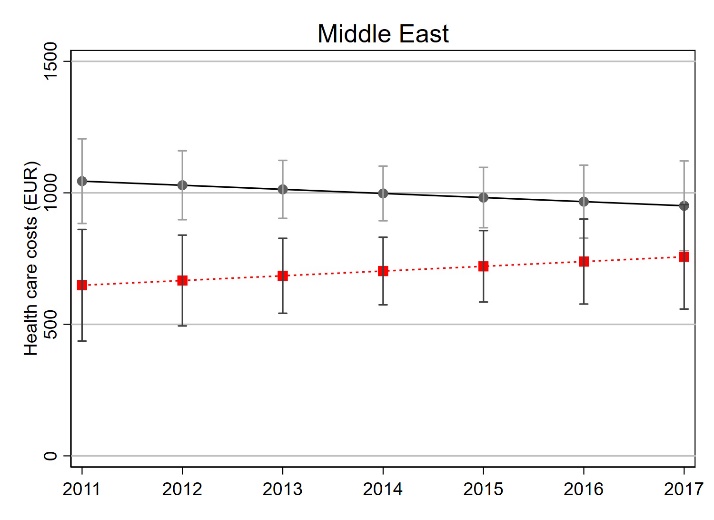

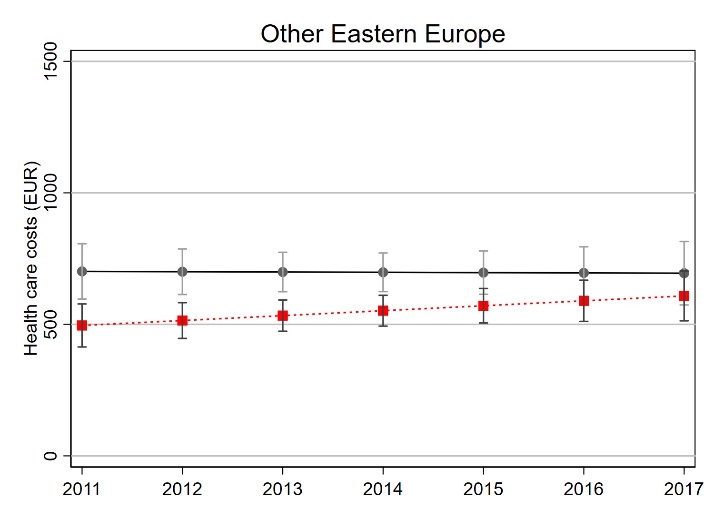


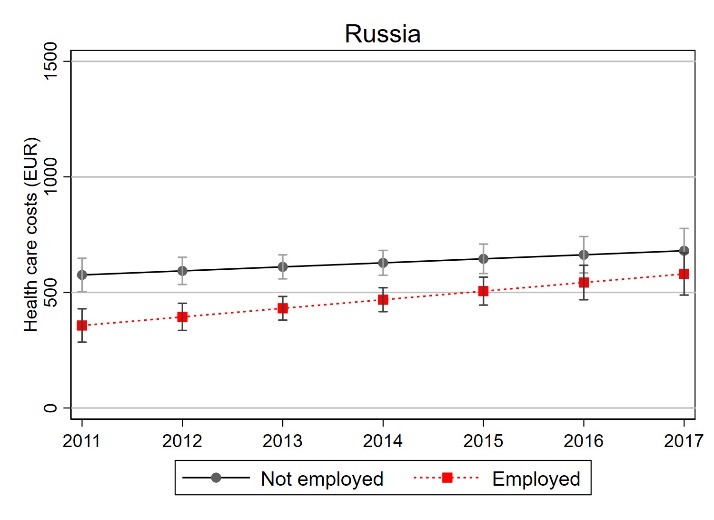

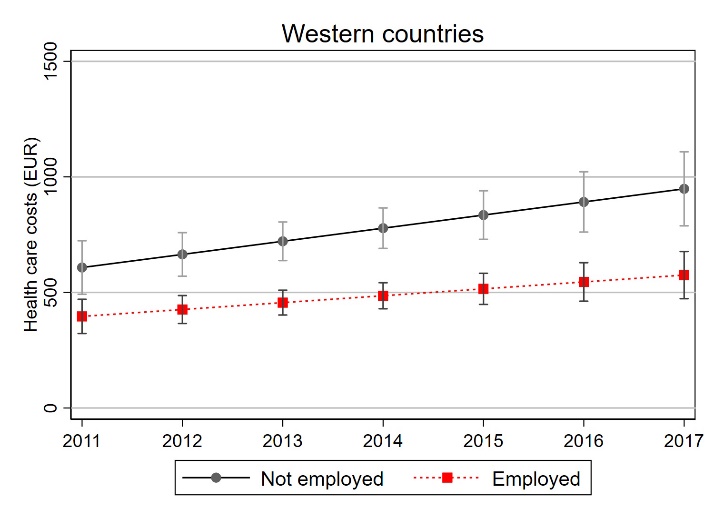


Note: Model controls for age, gender, region of residence, annual taxable income, and number of children in the household. Costs are deflated to the price level of 2020. Models are run separately by country.

**Appendix Figure A3. Total health care costs by income status, separately by country of origin.**


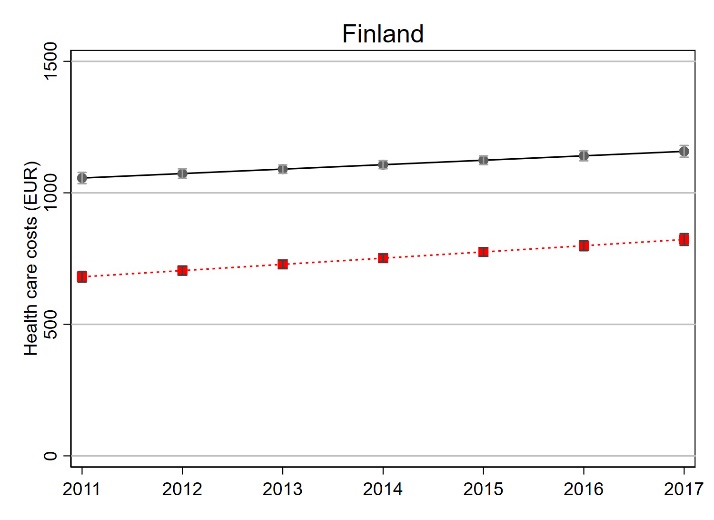

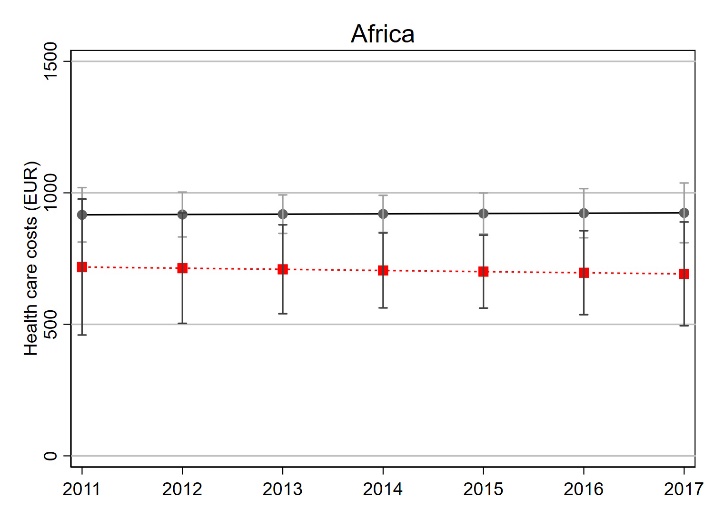


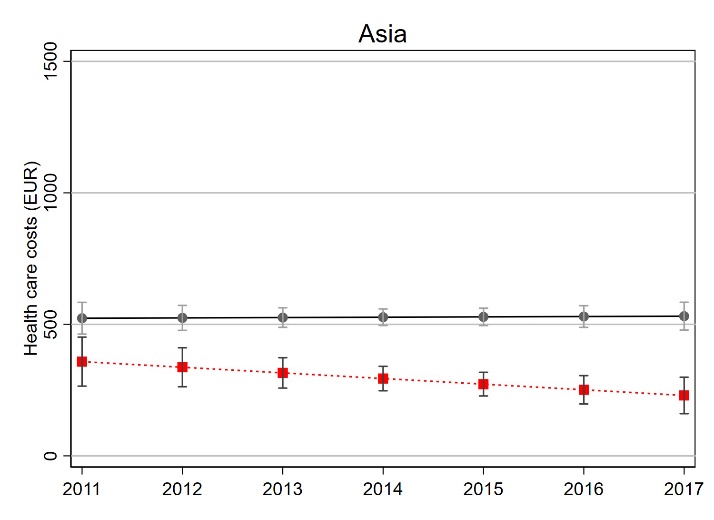

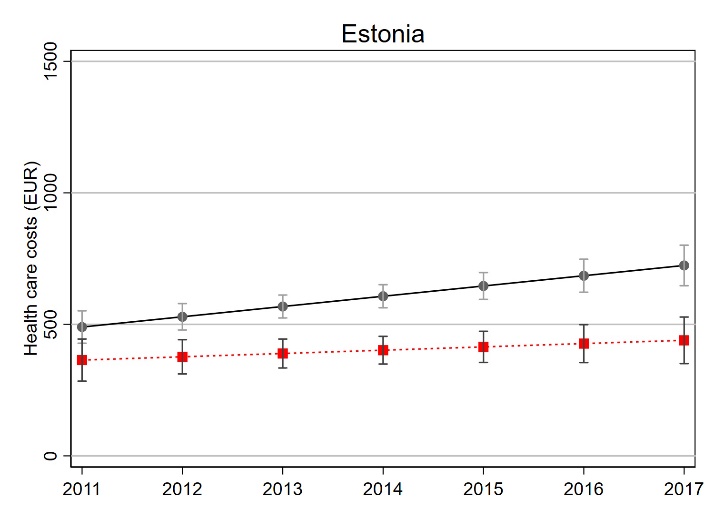


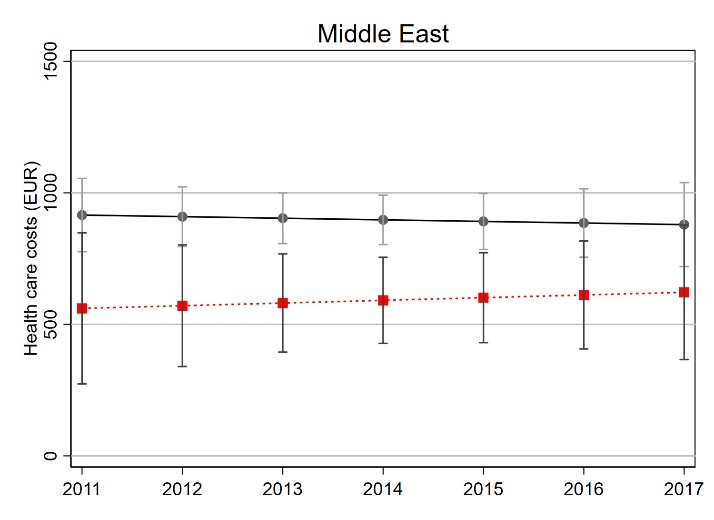

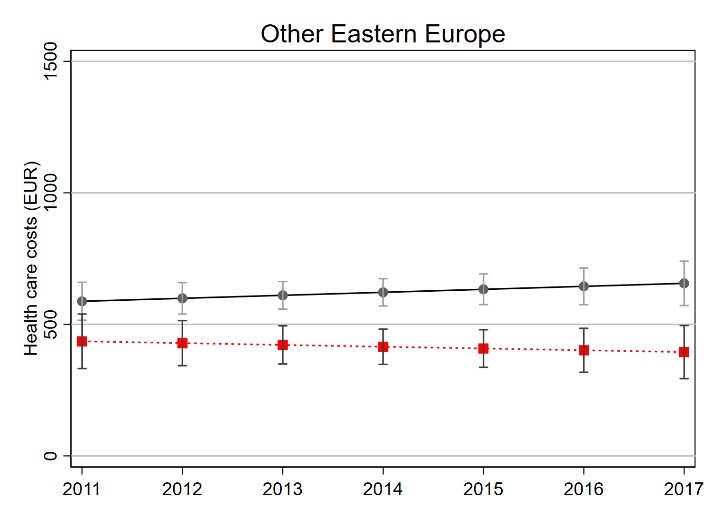


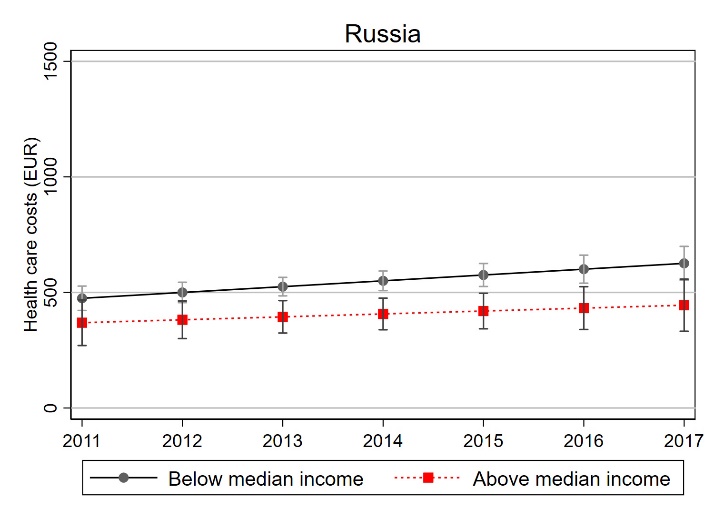

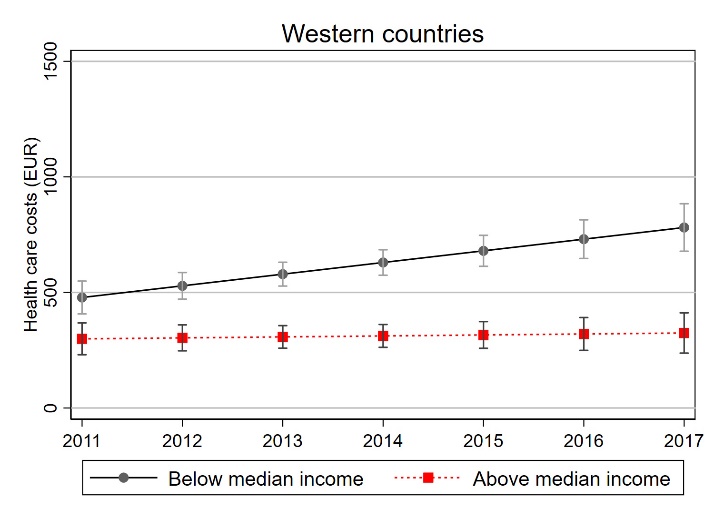


Note: Relative income status refers to income lower than the median of native-born Finns in the sample and the year in question and equal or higher than the median. Model controls for age, gender, region of residence, employment, and number of children in the household. Costs are deflated to the price level of 2020. Models are run separately by country.

**Appendix Table A1. Regression results (regarding Figures 2 and 3).**

|  | **Health care use, model A** | **Health care use, model B** | **Health care costs, model A** | **Health care costs, model B** |
| --- | --- | --- | --- | --- |
|  |  |  |  |  |
| Time | 0.005^***^ | 0.006^***^ | 26.463^***^ | 27.008^***^ |
|  | (0.000) | (0.000) | (1.854) | (1.890) |
| **Country of origin (ref. Finland)** |  |  |  |  |
| Western countries | -0.086^***^ | -0.058^***^ | -364.168^***^ | -419.768^***^ |
|  | (0.009) | (0.010) | (86.729) | (97.887) |
|  |  |  |  |  |
| Russia/USSR | -0.094^***^ | -0.113^***^ | -358.106^***^ | -609.539^***^ |
|  | (0.006) | (0.006) | (62.550) | (65.540) |
|  |  |  |  |  |
| Estonia | -0.102^***^ | -0.093^***^ | -383.274^***^ | -454.331^***^ |
|  | (0.006) | (0.006) | (61.667) | (64.448) |
|  |  |  |  |  |
| Other Eastern Europe | -0.048^***^ | -0.051^***^ | -256.487^**^ | -421.896^***^ |
|  | (0.008) | (0.008) | (81.277) | (85.708) |
|  |  |  |  |  |
| Middle East | 0.081^***^ | 0.051^***^ | 211.732^**^ | -153.976^*^ |
|  | (0.007) | (0.007) | (66.901) | (69.630) |
|  |  |  |  |  |
| Asia | -0.102^***^ | -0.101^***^ | -260.062^***^ | -438.734^***^ |
|  | (0.006) | (0.006) | (56.343) | (60.861) |
|  |  |  |  |  |
| Africa | 0.077^***^ | 0.054^***^ | 232.450^**^ | -89.981 |
|  | (0.007) | (0.008) | (74.632) | (77.203) |
| **Country of origin (ref. Finland) ## time** |  |  |  |  |
| Western countries | -0.002 | 0.004 | -5.428 | 20.976 |
|  | (0.002) | (0.002) | (17.460) | (20.140) |
|  |  |  |  |  |
| Russia/USSR | 0.001 | 0.004^**^ | -11.861 | 4.974 |
|  | (0.001) | (0.001) | (12.595) | (13.275) |
|  |  |  |  |  |
| Estonia | 0.003^*^ | 0.005^***^ | 0.089 | 8.810 |
|  | (0.001) | (0.001) | (12.422) | (13.119) |
|  |  |  |  |  |
| Other Eastern Europe | -0.000 | 0.003 | -14.037 | 1.673 |
|  | (0.002) | (0.002) | (16.363) | (17.521) |
|  |  |  |  |  |
| Middle East | -0.008^***^ | -0.003^*^ | -41.443^**^ | -24.076 |
|  | (0.001) | (0.001) | (13.444) | (14.144) |
|  |  |  |  |  |
| Asia | -0.005^***^ | 0.000 | -39.377^***^ | -17.601 |
|  | (0.001) | (0.001) | (11.325) | (12.518) |
|  |  |  |  |  |
| Africa | -0.009^***^ | -0.005^***^ | -42.480^**^ | -27.328 |
|  | (0.001) | (0.002) | (15.010) | (15.736) |
|  |  |  |  |  |
| Age | 0.001^***^ | 0.002^***^ | 9.134^***^ | 13.640^***^ |
|  | (0.000) | (0.000) | (0.478) | (0.500) |
|  |  |  |  |  |
| Female | 0.110^***^ | 0.105^***^ | 216.160^***^ | 156.538^***^ |
|  | (0.001) | (0.001) | (11.190) | (11.508) |
|  |  |  |  |  |
| Number of children |  | 0.009^***^ |  | 27.886^***^ |
|  |  | (0.000) |  | (4.696) |
|  |  |  |  |  |
| Employed |  | -0.056^***^ |  | -526.053^***^ |
|  |  | (0.001) |  | (9.516) |
|  |  |  |  |  |
| Personal annual income |  | -0.000^***^ |  | -0.008^***^ |
|  |  | (0.000) |  | (0.000) |
|  |  |  |  |  |
| Constant | 0.207^***^ | 0.233^***^ | 331.988^***^ | 819.048^***^ |
|  | (0.002) | (0.002) | (20.749) | (23.954) |
| *Observations* | *2,070,432* | *2,004,521* | *2,070,432* | *2,004,521* |

Standard errors in parentheses

^*^ *p* < 0.05, ^**^ *p* < 0.01, ^***^ *p* < 0.001

Note: Also controlled for region of residence.

**Appendix Table A2. Regression results (regarding Figures 4 and 5).**

|  | **Health care costs, model A, users** | **Health care costs, model B, users** | **Health care costs, men** | **Health care costs, women** |
| --- | --- | --- | --- | --- |
|  |  |  |  |  |
| Time | 9.068 | 3.538 | 20.028^***^ | 32.239^***^ |
|  | (5.089) | (5.124) | (2.855) | (2.473) |
| **Country of origin (ref. Finland)** |  |  |  |  |
| Western countries | -687.385^*^ | -957.902^***^ | -492.734^***^ | -245.980 |
|  | (284.270) | (290.904) | (130.594) | (154.132) |
|  |  |  |  |  |
| Russia/USSR | -458.316^*^ | -1113.836^***^ | -566.584^***^ | -638.844^***^ |
|  | (190.740) | (192.775) | (116.725) | (73.308) |
|  |  |  |  |  |
| Estonia | -372.362 | -644.971^***^ | -451.429^***^ | -462.634^***^ |
|  | (193.738) | (194.757) | (103.190) | (78.012) |
|  |  |  |  |  |
| Other Eastern Europe | -401.199 | -900.801^***^ | -481.901^***^ | -373.812^***^ |
|  | (237.762) | (239.696) | (130.292) | (109.554) |
|  |  |  |  |  |
| Middle East | 52.055 | -867.660^***^ | -304.823^***^ | 248.934^*^ |
|  | (170.136) | (172.156) | (92.558) | (110.884) |
|  |  |  |  |  |
| Asia | 36.537 | -661.570^***^ | -405.517^***^ | -482.371^***^ |
|  | (182.656) | (185.028) | (104.004) | (70.068) |
|  |  |  |  |  |
| Africa | 157.781 | -701.908^***^ | -313.626^**^ | 233.455^*^ |
|  | (190.632) | (192.108) | (110.345) | (107.018) |
|  |  |  |  |  |
| Other/unknown | -722.698^*^ | -1242.300^***^ | -548.849^**^ | -373.133^*^ |
|  | (350.968) | (353.387) | (181.740) | (167.158) |
| **Country of origin (ref. Finland) ## time** |  |  |  |  |
| Western countries | 52.827 | 84.115 | 26.299 | 15.933 |
|  | (60.580) | (61.858) | (25.927) | (33.415) |
|  |  |  |  |  |
| Russia/USSR | -3.967 | 47.432 | 3.747 | 5.994 |
|  | (40.415) | (40.753) | (22.903) | (15.668) |
|  |  |  |  |  |
| Estonia | -2.371 | 19.734 | 5.060 | 13.075 |
|  | (40.850) | (41.022) | (20.343) | (16.757) |
|  |  |  |  |  |
| Other Eastern Europe | -15.200 | 23.602 | 8.953 | -4.152 |
|  | (50.439) | (50.797) | (25.765) | (23.597) |
|  |  |  |  |  |
| Middle East | -52.485 | -20.459 | 8.934 | -97.714^***^ |
|  | (36.404) | (36.743) | (18.119) | (23.791) |
|  |  |  |  |  |
| Asia | -78.970^*^ | -9.857 | -22.479 | -12.483 |
|  | (39.237) | (39.637) | (20.811) | (15.141) |
|  |  |  |  |  |
| Africa | -41.774 | -4.934 | -13.978 | -44.725 |
|  | (40.954) | (41.284) | (21.754) | (22.947) |
|  |  |  |  |  |
| Other/unknown | 42.559 | 87.763 | 8.012 | 17.750 |
|  | (74.413) | (74.834) | (36.145) | (35.973) |
|  |  |  |  |  |
| Age | 18.076^***^ | 24.108^***^ | 21.742^***^ | 6.234^***^ |
|  | (1.019) | (1.067) | (0.789) | (0.608) |
| Female | -67.229^**^ | -135.536^***^ |  |  |
|  | (24.358) | (24.841) |  |  |
|  |  |  |  |  |
| Number of children |  | -7.931 | -72.923^***^ | 100.080^***^ |
|  |  | (10.869) | (7.282) | (5.852) |
|  |  |  |  |  |
| Employed |  | -1218.106^***^ | -485.194^***^ | -563.159^***^ |
|  |  | (25.368) | (14.255) | (12.589) |
|  |  |  |  |  |
| Personal annual income |  | -0.015^***^ | -0.008^***^ | -0.007^***^ |
|  |  | (0.001) | (0.000) | (0.000) |
|  |  |  |  |  |
| Constant | 1710.396^***^ | 3004.461^***^ | 576.254^***^ | 1183.051^***^ |
|  | (47.818) | (55.636) | (36.437) | (28.812) |
| Observations | 671,333 | 660,806 | 1,011,633 | 992,888 |

Standard errors in parentheses

^*^ *p* < 0.05, ^**^ *p* < 0.01, ^***^ *p* < 0.001

Note: Also controlled for region of residence.

**Appendix Table A3. Regression results (regarding Figure 6).**

|  |  |  |  |
| --- | --- | --- | --- |
|  | **Non-Finn partner** | **Finnish partner** | **No partner** |
|  |  |  |  |
| Time | 38.247^***^ | 30.387^***^ | 11.431^*^ |
|  | (9.586) | (1.734) | (5.755) |
| **Country of origin (ref. Finland)** |  |  |  |
| Western countries | -273.592 | -239.642^**^ | -938.435^*^ |
|  | (205.590) | (77.018) | (454.551) |
| Russia/USSR | -293.743^***^ | -464.582^***^ | -1149.694^***^ |
|  | (75.732) | (102.306) | (259.479) |
| Estonia | -274.766^***^ | -154.694 | -851.917^***^ |
|  | (78.324) | (124.279) | (183.686) |
| Other Eastern Europe | -177.430 | -273.864^*^ | -885.192^**^ |
|  | (91.621) | (129.188) | (340.193) |
| Middle East | 353.445^***^ | -292.855^**^ | -677.245^**^ |
|  | (84.318) | (105.368) | (218.576) |
| Asia | -78.105 | -375.516^***^ | -985.783^***^ |
|  | (79.632) | (68.327) | (240.408) |
| Africa | 312.611^**^ | -228.403 | -441.343^*^ |
|  | (95.123) | (120.991) | (214.347) |
| **Country of origin (ref. Finland) ## time** |  |  |  |
| Western countries | 5.096 | 8.082 | 78.313 |
|  | (42.887) | (18.384) | (88.194) |
| Russia/USSR | -25.490 | 14.754 | 43.413 |
|  | (15.379) | (24.187) | (48.635) |
| Estonia | 0.856 | -1.947 | 13.791 |
|  | (16.028) | (29.667) | (34.324) |
| Other Eastern Europe | -21.687 | 17.020 | 6.431 |
|  | (18.702) | (30.849) | (65.953) |
| Middle East | -87.054^***^ | -4.969 | 59.050 |
|  | (17.091) | (25.107) | (40.745) |
| Asia | -51.859^**^ | -15.256 | 19.775 |
|  | (16.316) | (16.275) | (46.989) |
| Africa | -71.640^***^ | 3.332 | -2.424 |
|  | (19.427) | (28.969) | (39.854) |
| Age | 6.113^***^ | 9.976^***^ | 24.322^***^ |
|  | (1.197) | (0.396) | (1.584) |
| Female | 376.236^***^ | 260.213^***^ | -37.304 |
|  | (23.057) | (9.053) | (40.075) |
| Number of children | 101.129^***^ | 37.127^***^ | -28.003 |
|  | (9.335) | (3.689) | (27.522) |
| Employed | -262.695^***^ | -508.255^***^ | -597.902^***^ |
|  | (21.819) | (9.357) | (27.437) |
| Personal annual income | -0.001^*^ | -0.005^***^ | -0.016^***^ |
|  | (0.001) | (0.000) | (0.001) |
| Constant | 347.314^***^ | 658.958^***^ | 1144.125^***^ |
|  | (69.377) | (19.817) | (72.707) |
|  |  |  |  |
| *Observations* | *142,860* | *1,384,969* | *476,692* |

Note: Also controlled for region of residence. Standard errors in parentheses, ^*^ *p* < 0.05, ^**^ *p* < 0.01, ^***^ *p* < 0.001.

**Appendix Table A4. Regression results (regarding Figure 7).**

|  | **Not employed** | **Employed** |
| --- | --- | --- |
| Time | 1.650 | 27.167^***^ |
|  | (6.551) | (1.307) |
| **Country of origin (ref. Finland)** |  |  |
| Western countries | -1041.698^***^ | -145.345^*^ |
|  | (283.323) | (62.564) |
| Russia/USSR | -1089.616^***^ | -248.192^***^ |
|  | (149.358) | (49.810) |
| Estonia | -926.451^***^ | -241.165^***^ |
|  | (202.386) | (39.541) |
| Other Eastern Europe | -893.338^***^ | -180.818^**^ |
|  | (222.089) | (57.709) |
| Middle East | -408.088^**^ | -24.263 |
|  | (140.030) | (64.760) |
| Asia | -856.421^***^ | -166.343^***^ |
|  | (152.194) | (42.670) |
| Africa | -269.782 | -76.648 |
|  | (163.849) | (64.949) |
| **Country of origin (ref. Finland) ## time** |  |  |
| Western countries | 62.567 | 2.609 |
|  | (63.057) | (14.612) |
| Russia/USSR | 2.586 | 6.290 |
|  | (31.602) | (11.387) |
| Estonia | 15.494 | 11.617 |
|  | (43.407) | (9.342) |
|  |  |  |
| Other Eastern Europe | 11.914 | 3.146 |
|  | (48.034) | (13.464) |
| Middle East | -28.443 | -13.706 |
|  | (29.754) | (14.250) |
| Asia | -26.973 | -15.504 |
|  | (33.476) | (9.815) |
| Africa | -40.376 | 6.615 |
|  | (34.268) | (14.831) |
| Age | 26.017^***^ | 8.969^***^ |
|  | (1.249) | (0.287) |
| Female | 146.237^***^ | 222.125^***^ |
|  | (31.706) | (6.345) |
| Number of children | -14.655 | 39.413^***^ |
|  | (14.440) | (2.810) |
| Personal annual income | -0.016^***^ | -0.004^***^ |
|  | (0.001) | (0.000) |
| Constant | 866.602^***^ | 154.418^***^ |
|  | (58.763) | (13.666) |
| *Observations* | *506,228* | *1,498,293* |

Note: Also controlled for region of residence. Standard errors in parentheses, ^*^ *p* < 0.05, ^**^ *p* < 0.01, ^***^ *p* < 0.001.

**Appendix Table A5. Regression results (regarding Figure 8).**

|  | **Below median income** | **Above median income** |
| --- | --- | --- |
|  |  |  |
| Time | 15.356^***^ | 19.778^***^ |
|  | (3.334) | (1.524) |
| **Country of origin (ref. Finland)** |  |  |
| Western countries | -667.514^***^ | -256.267^***^ |
|  | (152.617) | (62.863) |
| Russia/USSR | -737.226^***^ | -222.595^**^ |
|  | (89.130) | (68.951) |
| Estonia | -624.441^***^ | -205.498^***^ |
|  | (95.689) | (52.938) |
| Other Eastern Europe | -574.573^***^ | -253.052^***^ |
|  | (121.547) | (75.818) |
| Middle East | -217.830^*^ | -210.615^*^ |
|  | (90.503) | (95.986) |
| Asia | -557.478^***^ | -281.023^***^ |
|  | (81.867) | (57.387) |
| Africa | -150.207 | 66.282 |
|  | (100.489) | (112.287) |
| **Country of origin (ref. Finland) ## time** |  |  |
| Western countries | 40.243 | -18.843 |
|  | (32.759) | (14.105) |
| Russia/USSR | 3.661 | -11.856 |
|  | (18.275) | (15.253) |
| Estonia | 13.745 | -9.109 |
|  | (19.912) | (11.899) |
| Other Eastern Europe | 4.886 | -15.187 |
|  | (25.490) | (16.643) |
| Middle East | -27.386 | 9.264 |
|  | (18.617) | (19.931) |
| Asia | -17.139 | -34.783^**^ |
|  | (17.127) | (12.364) |
| Africa | -31.794 | -50.154^*^ |
|  | (20.770) | (22.768) |
| Age | 17.607^***^ | 8.118^***^ |
|  | (0.742) | (0.360) |
| Female | 136.108^***^ | 182.217^***^ |
|  | (18.564) | (7.517) |
| Number of children | 33.535^***^ | 6.136 |
|  | (7.823) | (3.394) |
| Employed | -564.823^***^ | -244.580^***^ |
|  | (12.613) | (11.196) |
| Constant | 722.618^***^ | 281.637^***^ |
|  | (35.214) | (19.784) |
| *Observations* | *1,060,315* | *1,010,117* |

Note: Also controlled for region of residence. Standard errors in parentheses, ^*^ *p* < 0.05, ^**^ *p* < 0.01, ^***^ *p* < 0.001.

**Appendix Table A6. Neighbourhood and workplace characteristics.**

| **Native share in the neighborhood less than 75%** | |
| --- | --- |
| Finnish | 0.6 % |
| Western countries | 2.1 % |
| Russia/USSR | 4.9 % |
| Estonia | 6.2 % |
| Other Eastern Europe | 7.4 % |
| Middle East | 6.8 % |
| Asia | 7.3 % |
| Africa | 9.4 % |
| Other/unknown | 5.8 % |
| **Native share in the workplace less than 75%** | |
| Finnish | 1.9 % |
| Western countries | 35.5 % |
| Russia/USSR | 48.6 % |
| Estonia | 55.5 % |
| Other Eastern Europe | 49.0 % |
| Middle East | 59.8 % |
| Asia | 56.3 % |
| Africa | 54.6 % |
| Other/unknown | 49.5 % |

**Appendix Table A7. Regression results (regarding Figure 9).**

|  | **Health care costs, model B** |
| --- | --- |
|  |  |
| Time | 25.130 |
|  | (13.194) |
|  |  |
| **Cohort (ref.1983–1989)** |  |
| Arrived 1990-1999 | -95.493 |
|  | (69.161) |
|  |  |
| Arrived 2001-2007 | -175.901^*^ |
|  | (69.092) |
|  |  |
| Arrived 2008-2010 | -264.335^***^ |
|  | (70.563) |
|  |  |
| Native-born Finns | 107.718 |
|  | (66.111) |
|  |  |
| **Cohort (ref.1983–1989) ## time** |  |
| Arrived 1990-1999 | 7.061 |
|  | (13.947) |
|  |  |
| Arrived 2001-2007 | -5.873 |
|  | (13.924) |
|  |  |
| Arrived 2008-2010 | -1.906 |
|  | (14.219) |
|  |  |
| Native-born Finns | 4.114 |
|  | (13.307) |
|  |  |
| Age | 10.462^***^ |
|  | (0.446) |
|  |  |
| Female | 138.363^***^ |
|  | (9.989) |
|  |  |
| Number of children | 19.981^***^ |
|  | (3.959) |
|  |  |
| Employed | -468.027^***^ |
|  | (8.029) |
| Personal annual income | -0.007^***^ |
|  | (0.000) |
|  |  |
| Constant | 759.914^***^ |
|  | (68.893) |
| Observations | 2,641,519 |

Standard errors in parentheses

^*^ *p* < 0.05, ^**^ *p* < 0.01, ^***^ *p* < 0.001

Note: Also controlled for region of residence.
